# Supplementary figures and images for: Doxorubicin-induced p53 interferes with mitophagy in cardiac fibroblasts
Source: PLoS One. 2020 Sep 22;15(9):e0238856. doi: 10.1371/journal.pone.0238856 (PMC7508395; doi:10.1371/journal.pone.0238856)

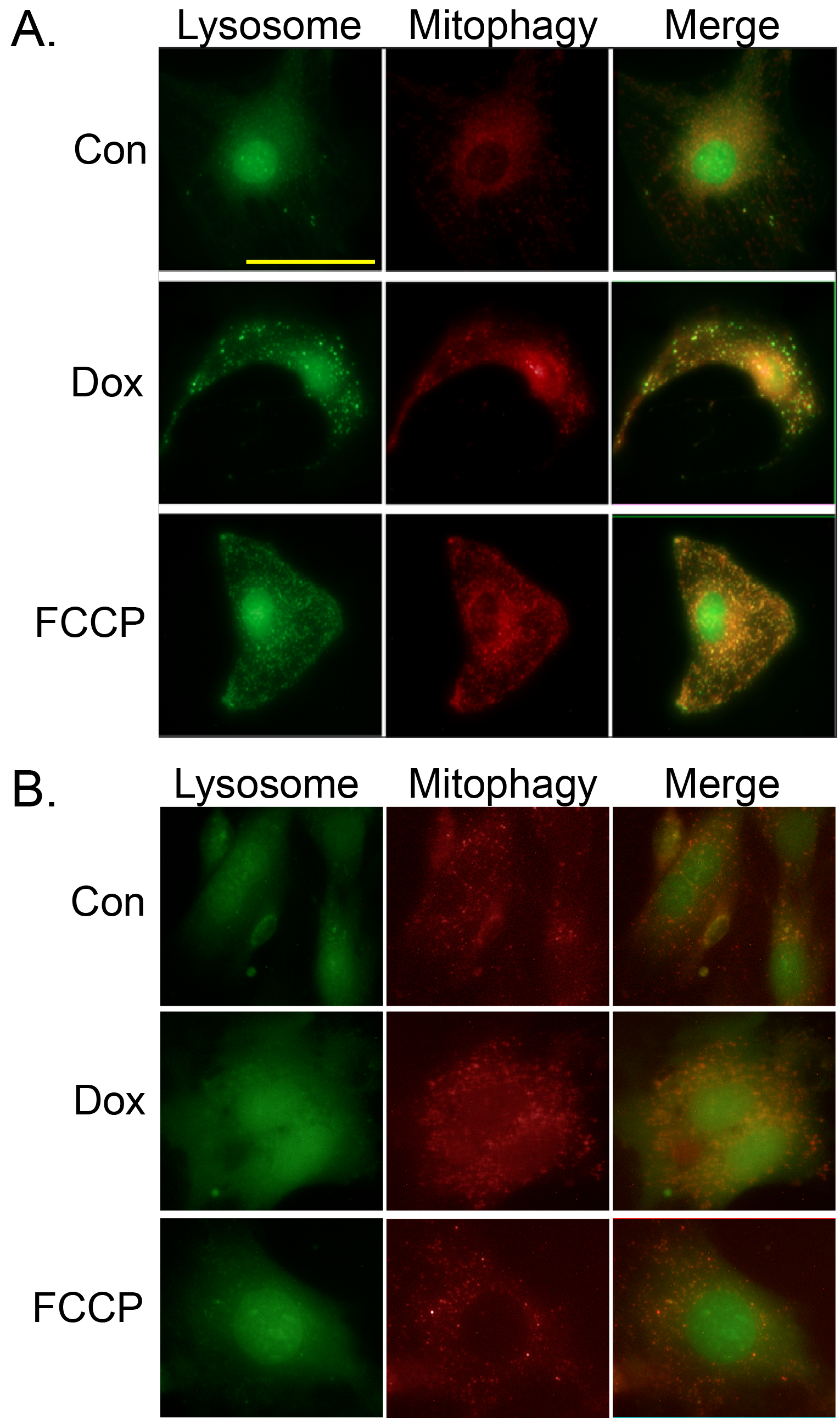

Supplement: S1 Fig — WT (A) and p53-/- (B) cells were stained with a mitochondrial dye before treatment and a lysosomal dye after treatment. Fluorescent intensity of the mitochondrial dye increases when it is exposed to an acidic pH. Magnification 60X. (TIF) [file pone.0238856.s001.tif]

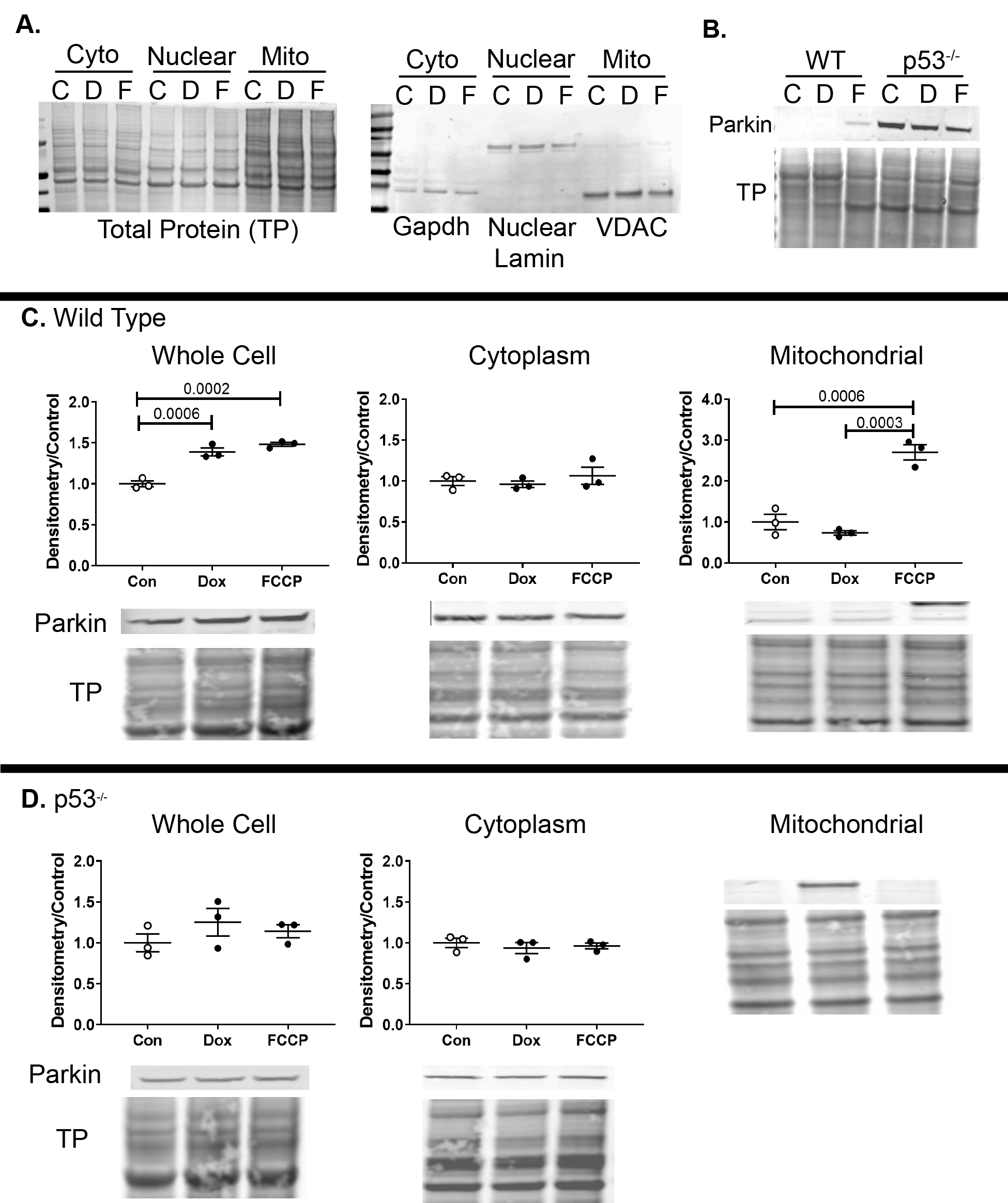

Supplement: S2 Fig — (A) Fraction specificity confirmed with subcellular specific antibodies. (B) Parkin expression, via WB, in WT and p53-/- after standard treatment. In (C) and (D), cells underwent standard treatment and at protein extraction separated into subcellular fractions according to kit protocol. The fractions were then separated using gel electrophoresis and probed for the presence of Parkin. Parkin was observed in the mitochondrial fraction after DOX exposure. Quantification include 3 biological replicates and is represented as average ± SEM. (TIF) [file pone.0238856.s002.tif]
