# Supplementary material for: Doxorubicin-induced p53 interferes with mitophagy in cardiac fibroblasts
Source: PLoS One. 2020 Sep 22;15(9):e0238856. doi: 10.1371/journal.pone.0238856 (PMC7508395; doi:10.1371/journal.pone.0238856)
Supplement: S1 Raw images — (PDF) [file pone.0238856.s003.pdf]

Fig 4A p53

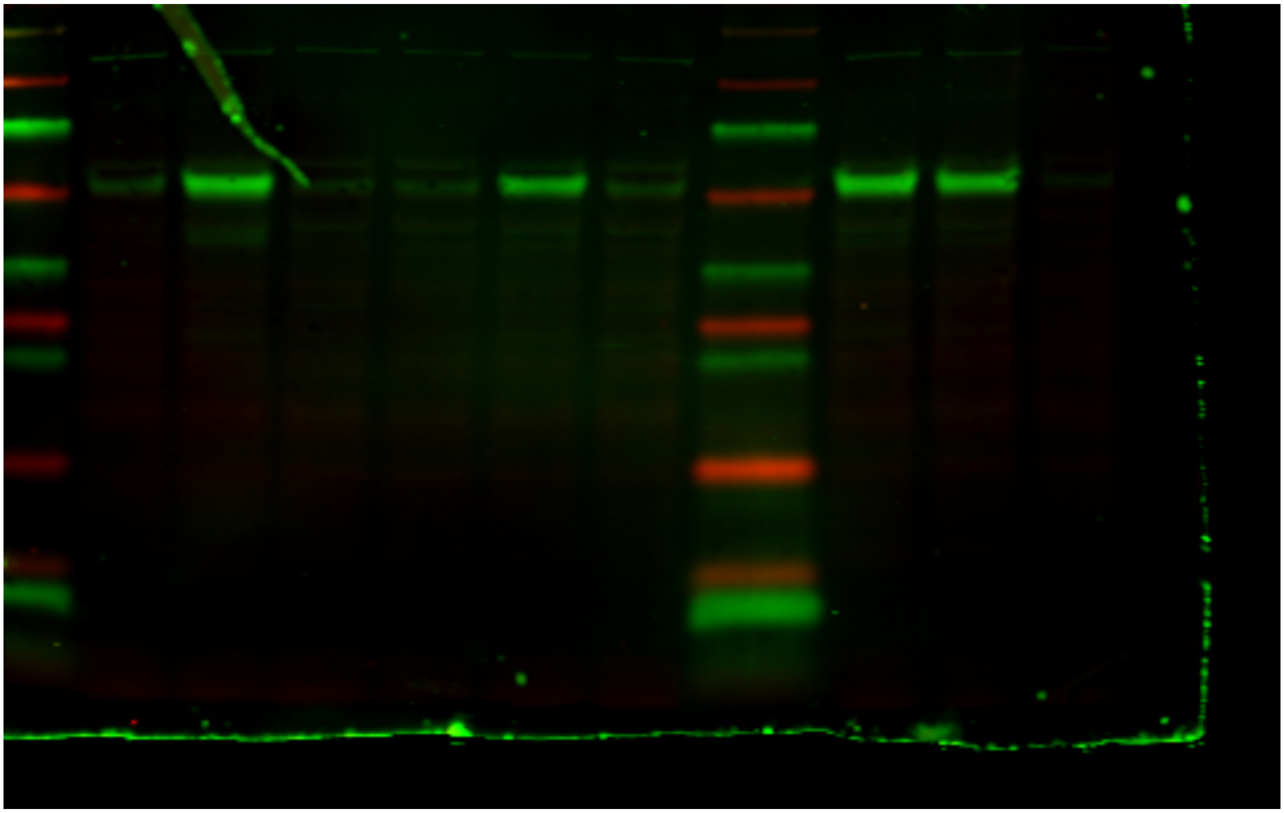

Fig 4A total protein

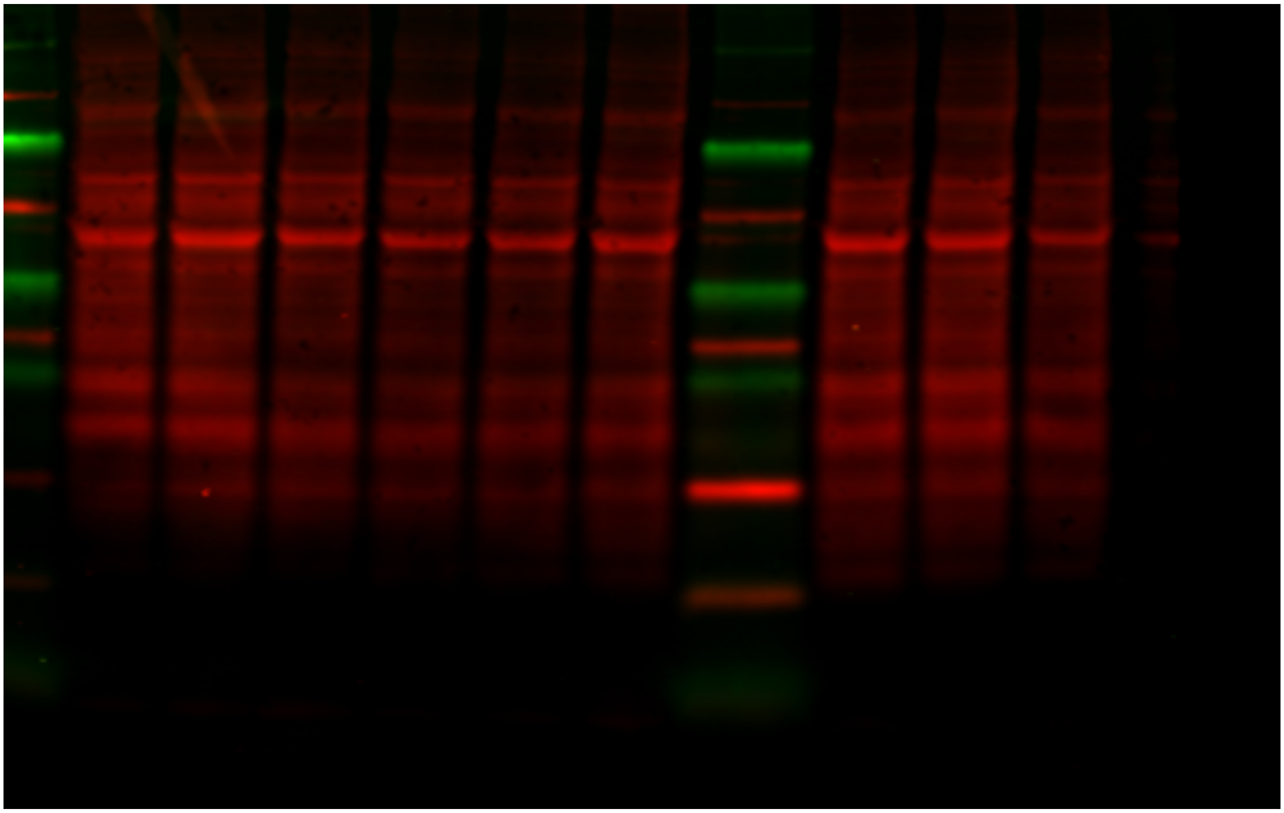

Fig 4C p53

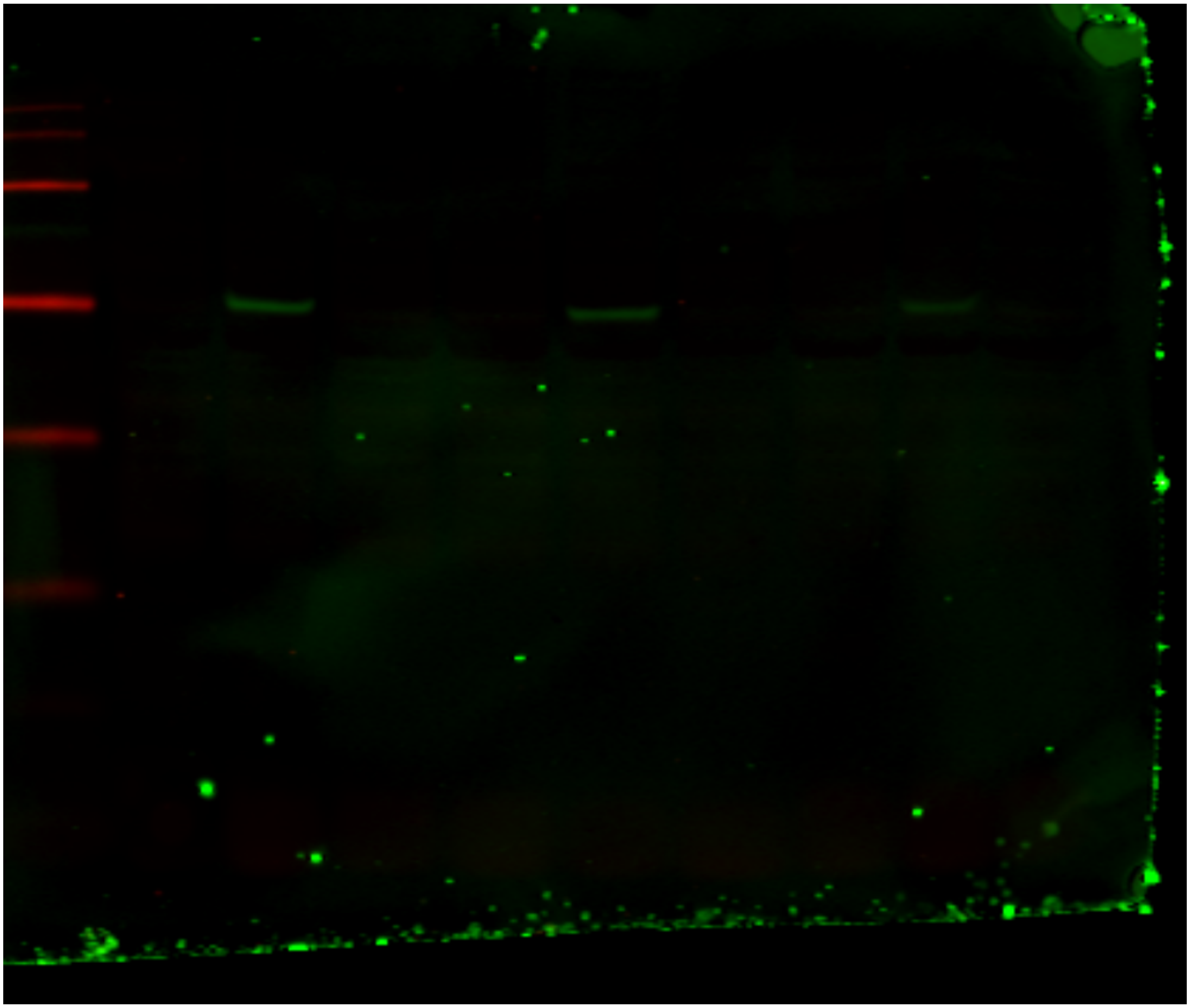

Fig 4C total protein

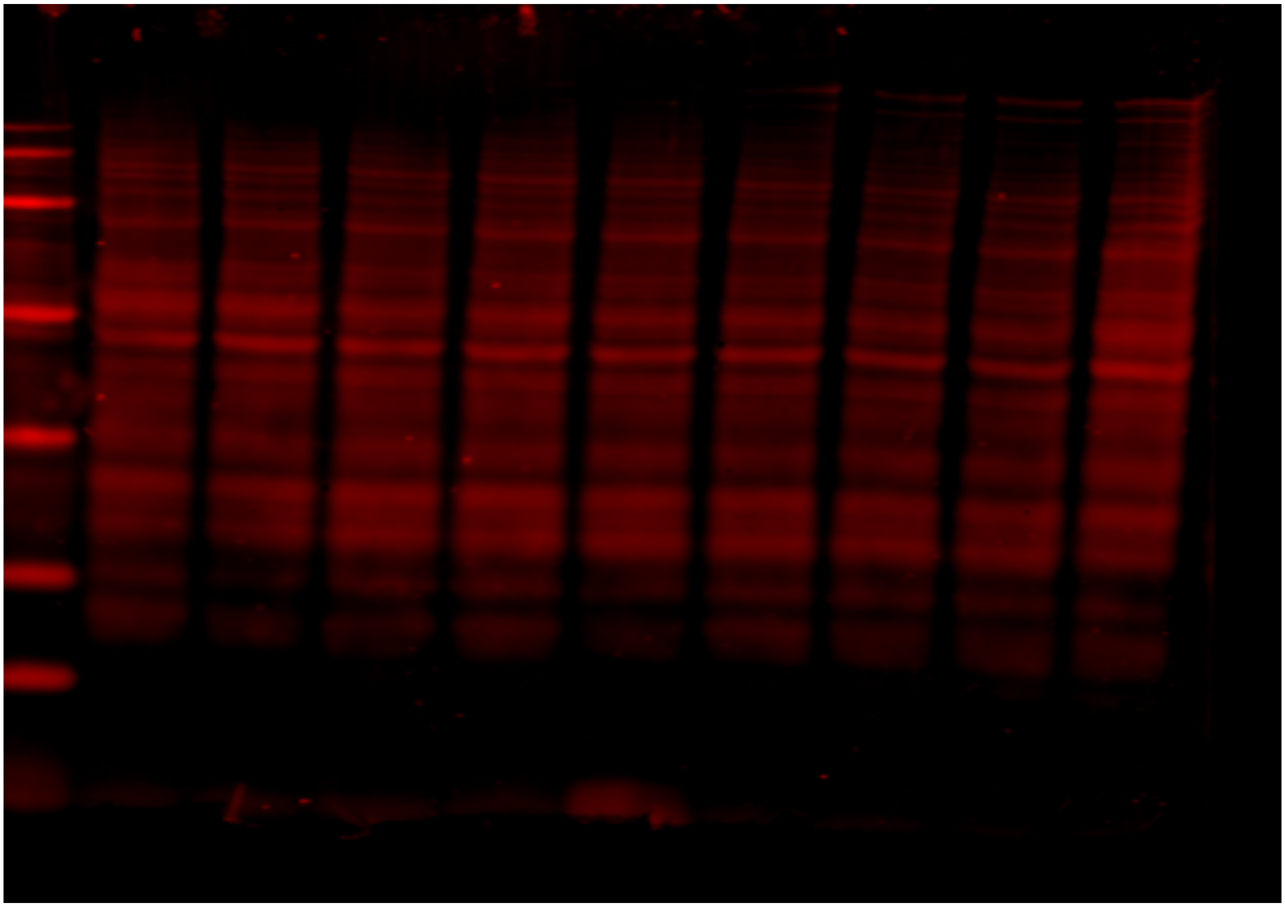

Fig 4F PGC1a

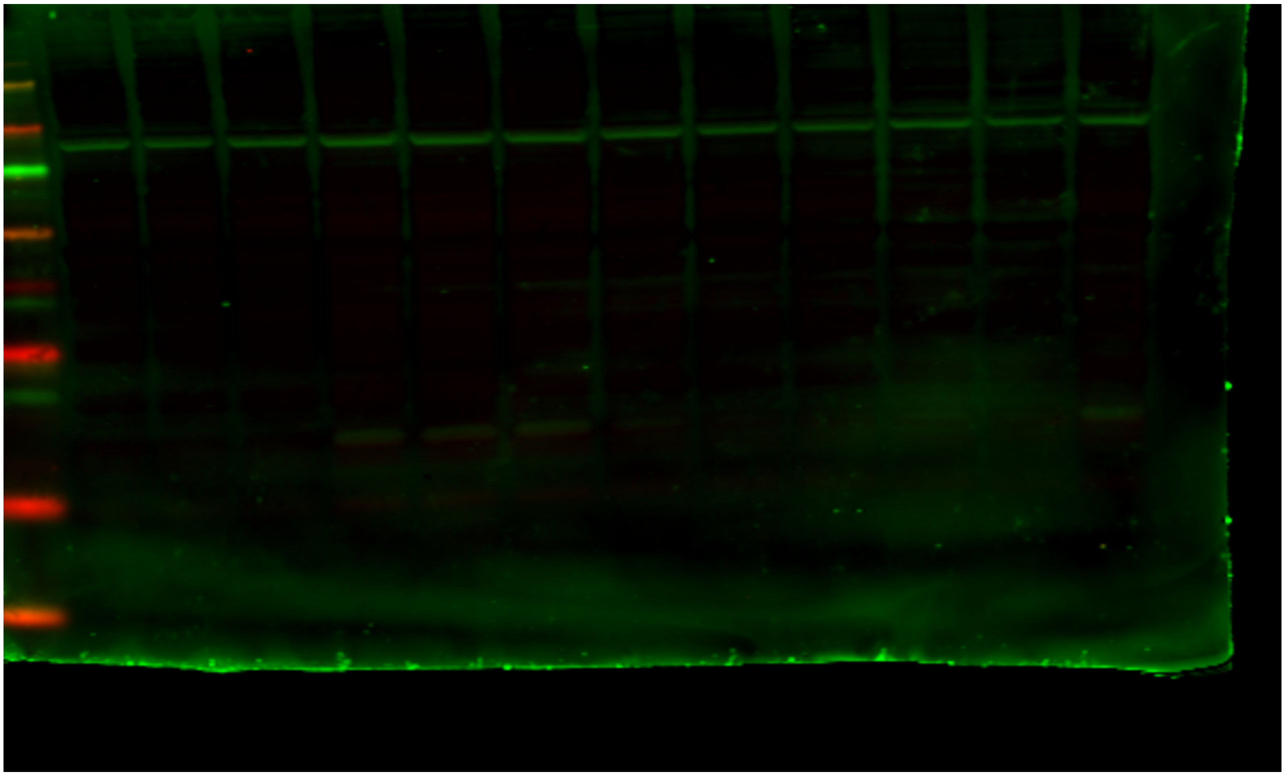

Fig 4F total protein

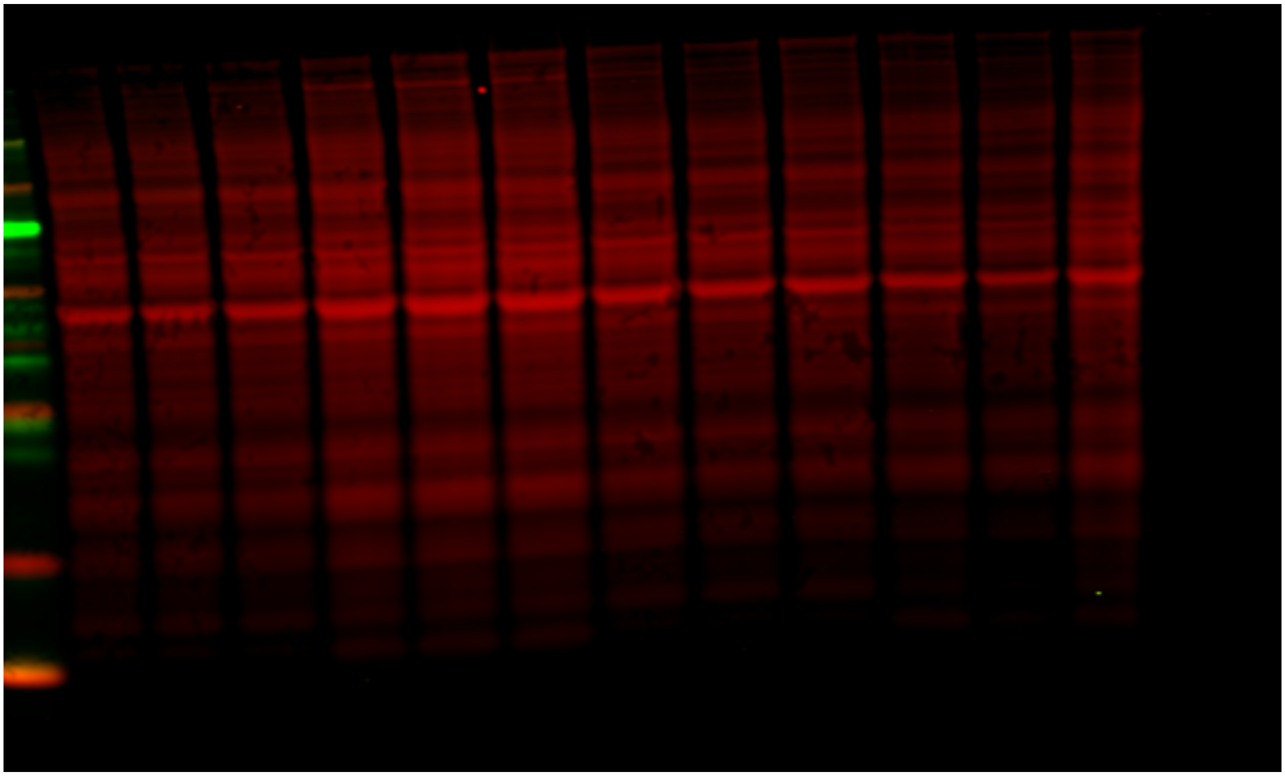

Fig 4G PGC1a

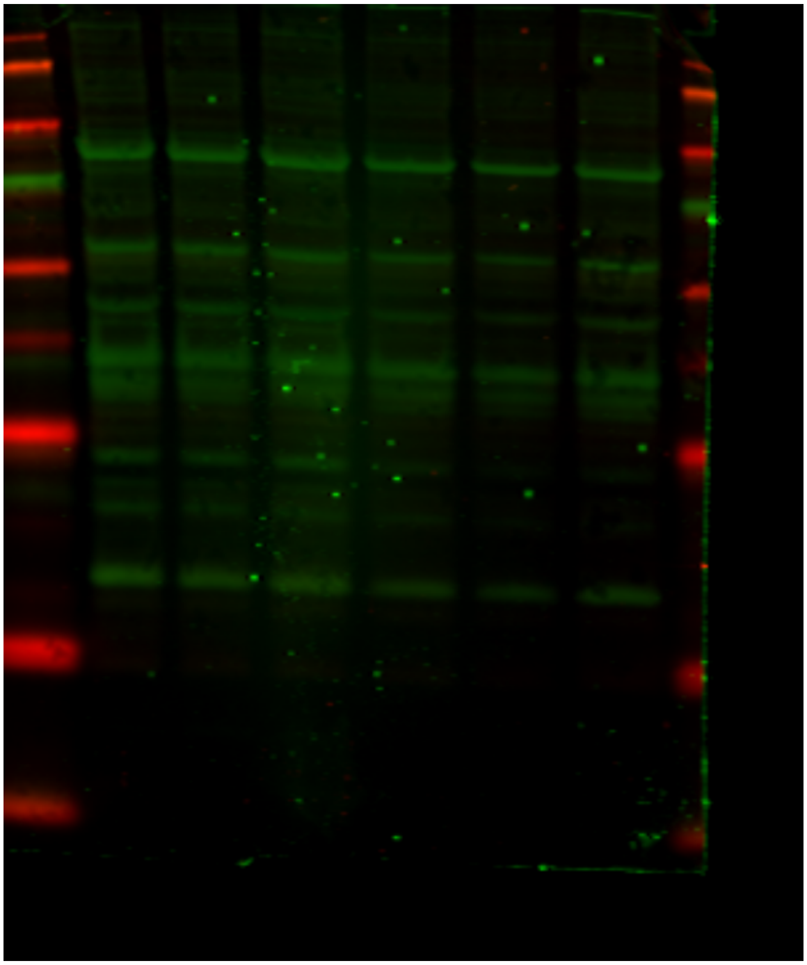

Fig 4G total protein

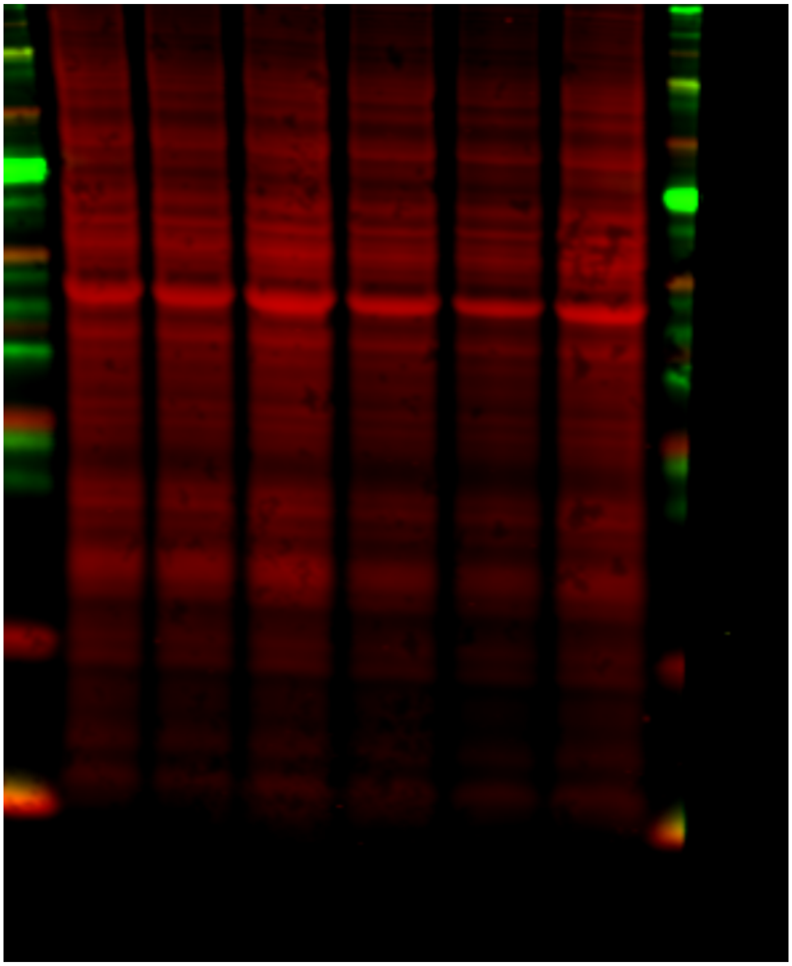

Fig S2A gapdh nuc lam VDAC

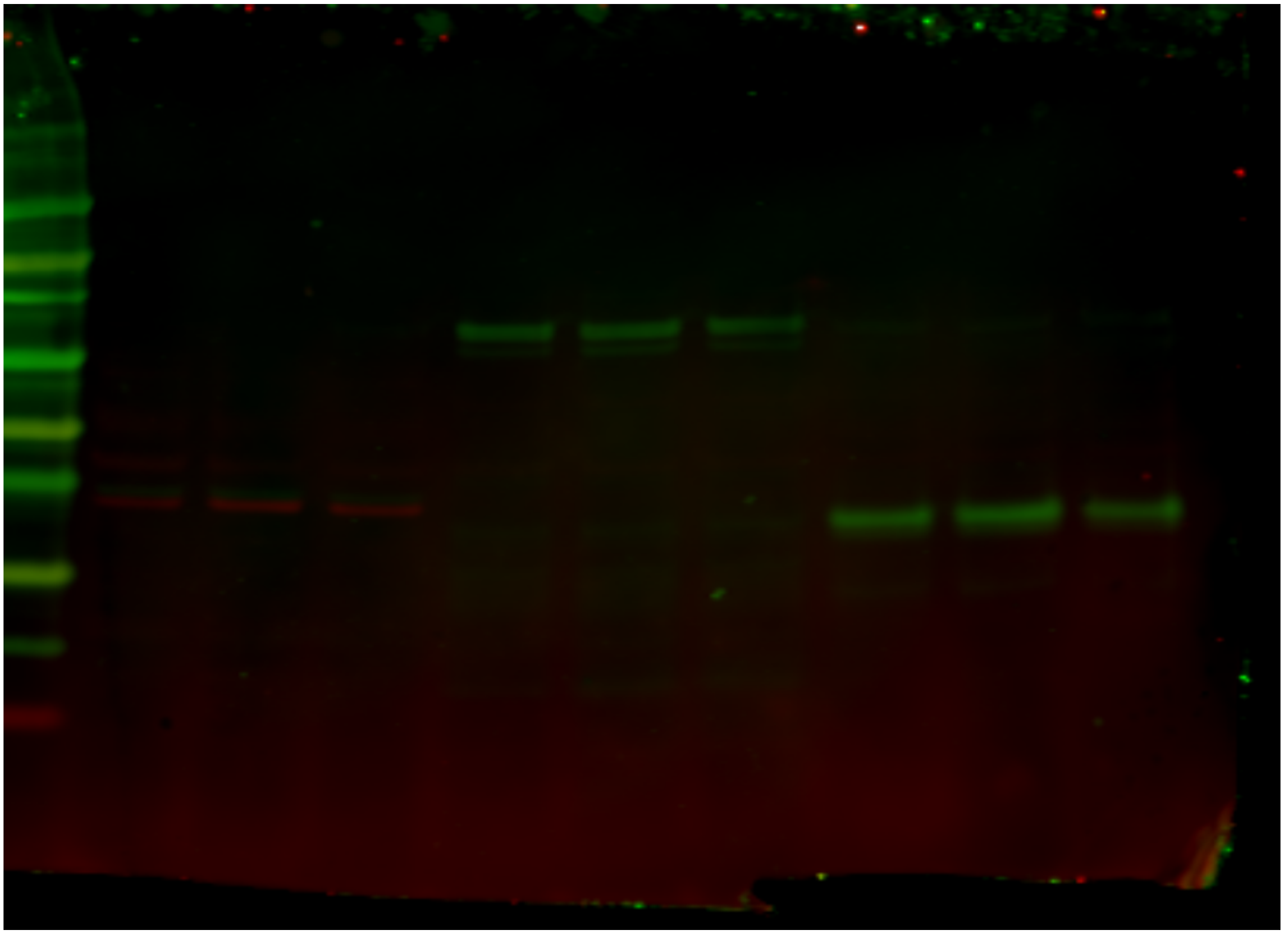

Fig S2A total protein

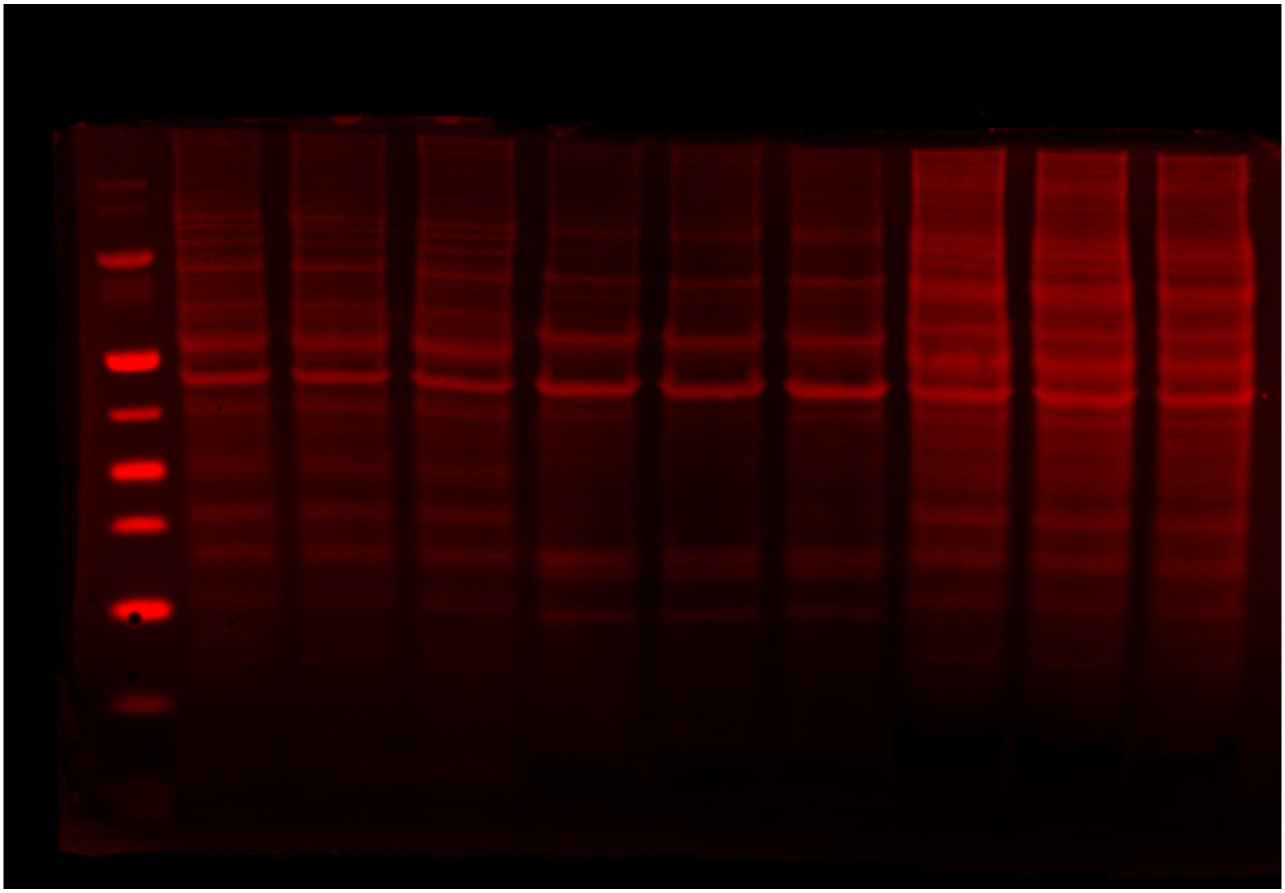

Fig S2B Parkin

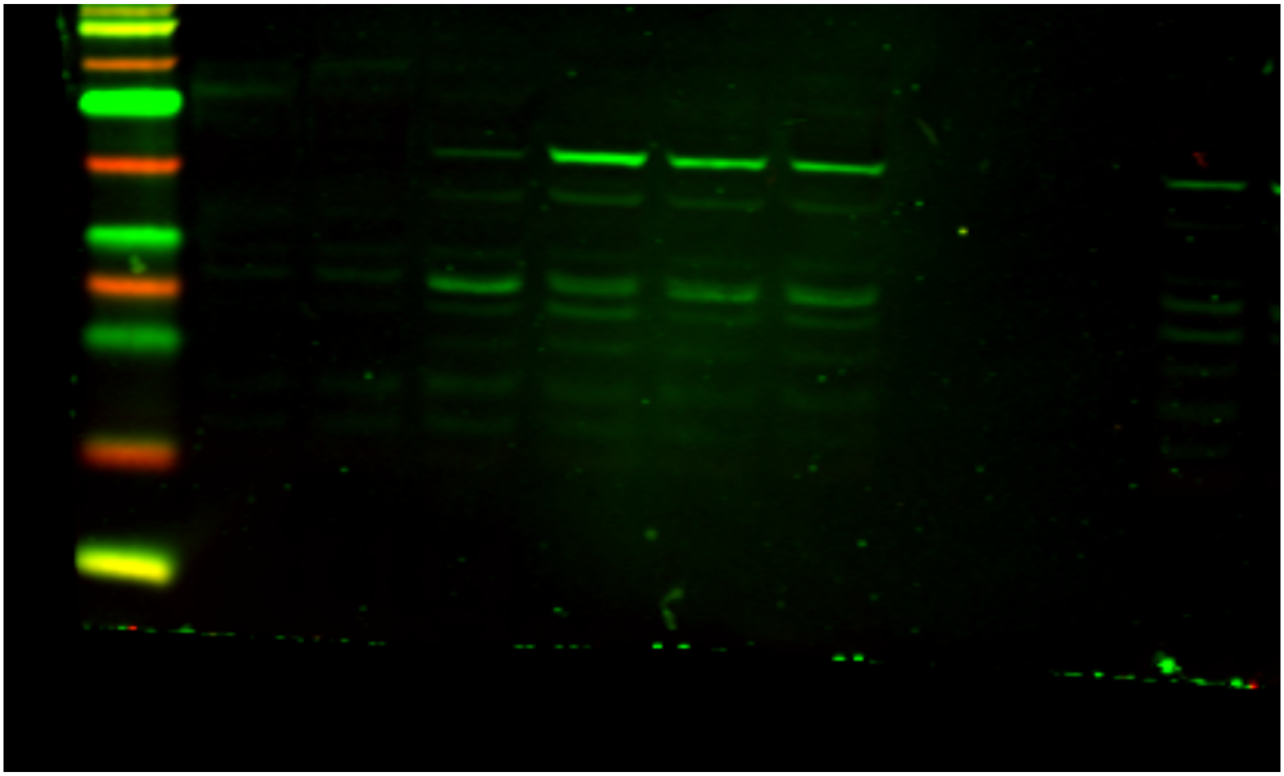

Fig S2B total protein

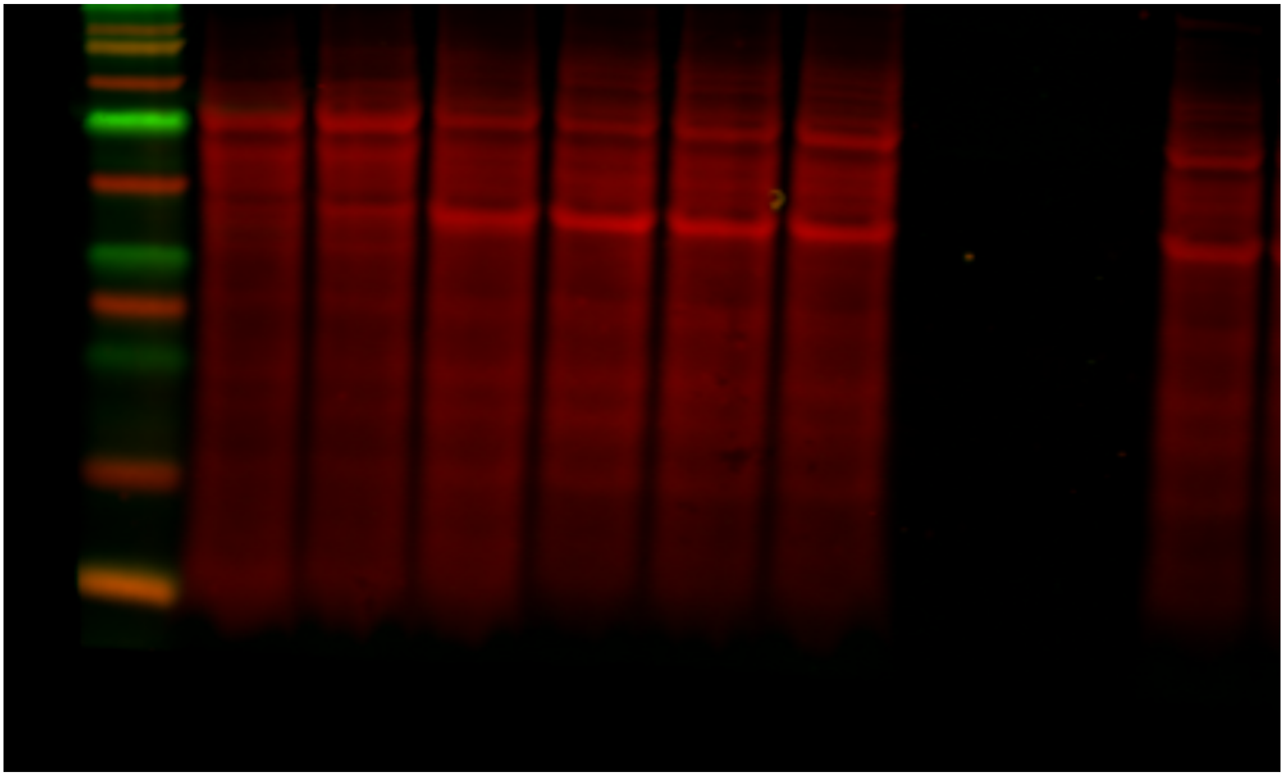

Fig S2C Cytoplasm Parkin

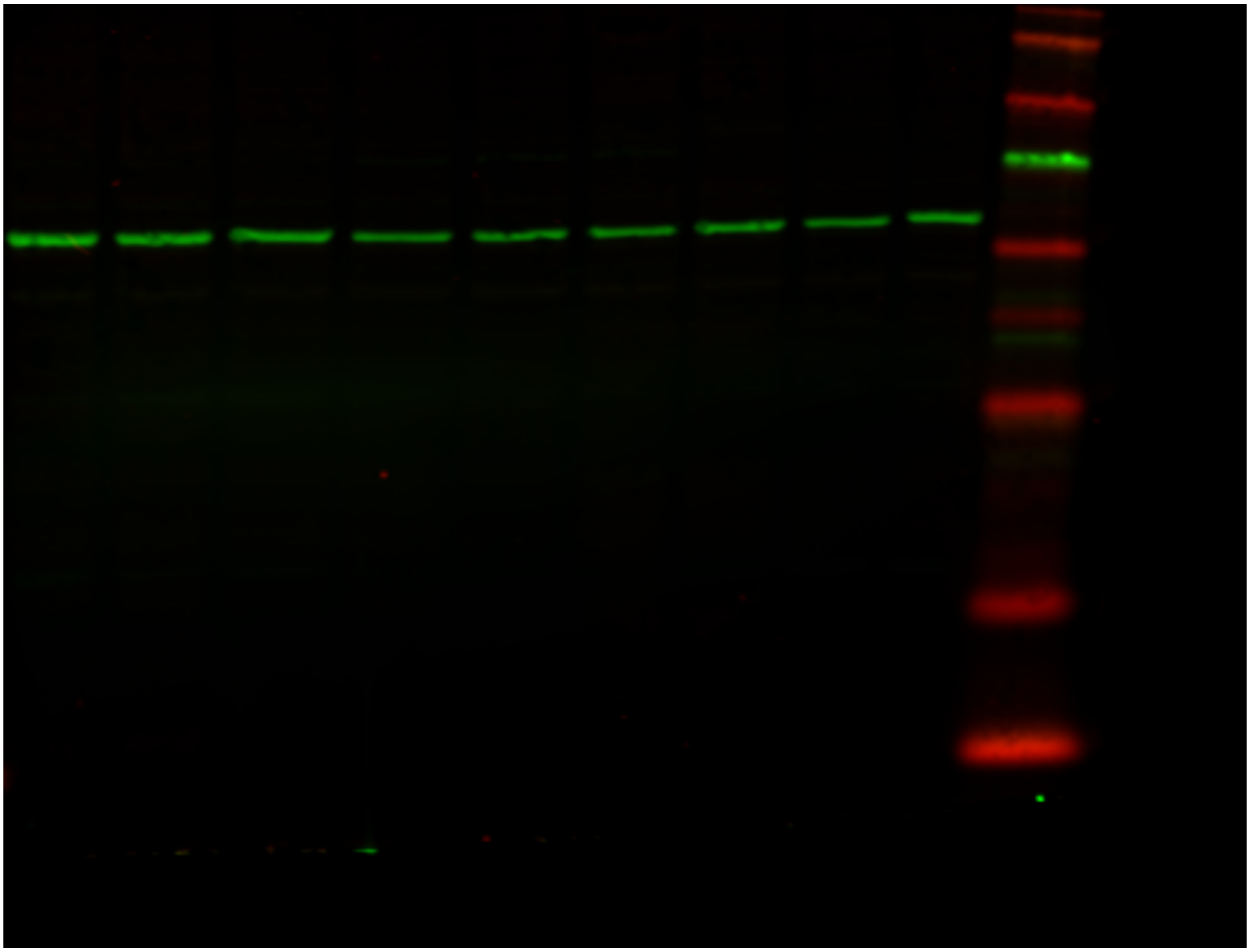

Fig S2C Cytoplasm total protein

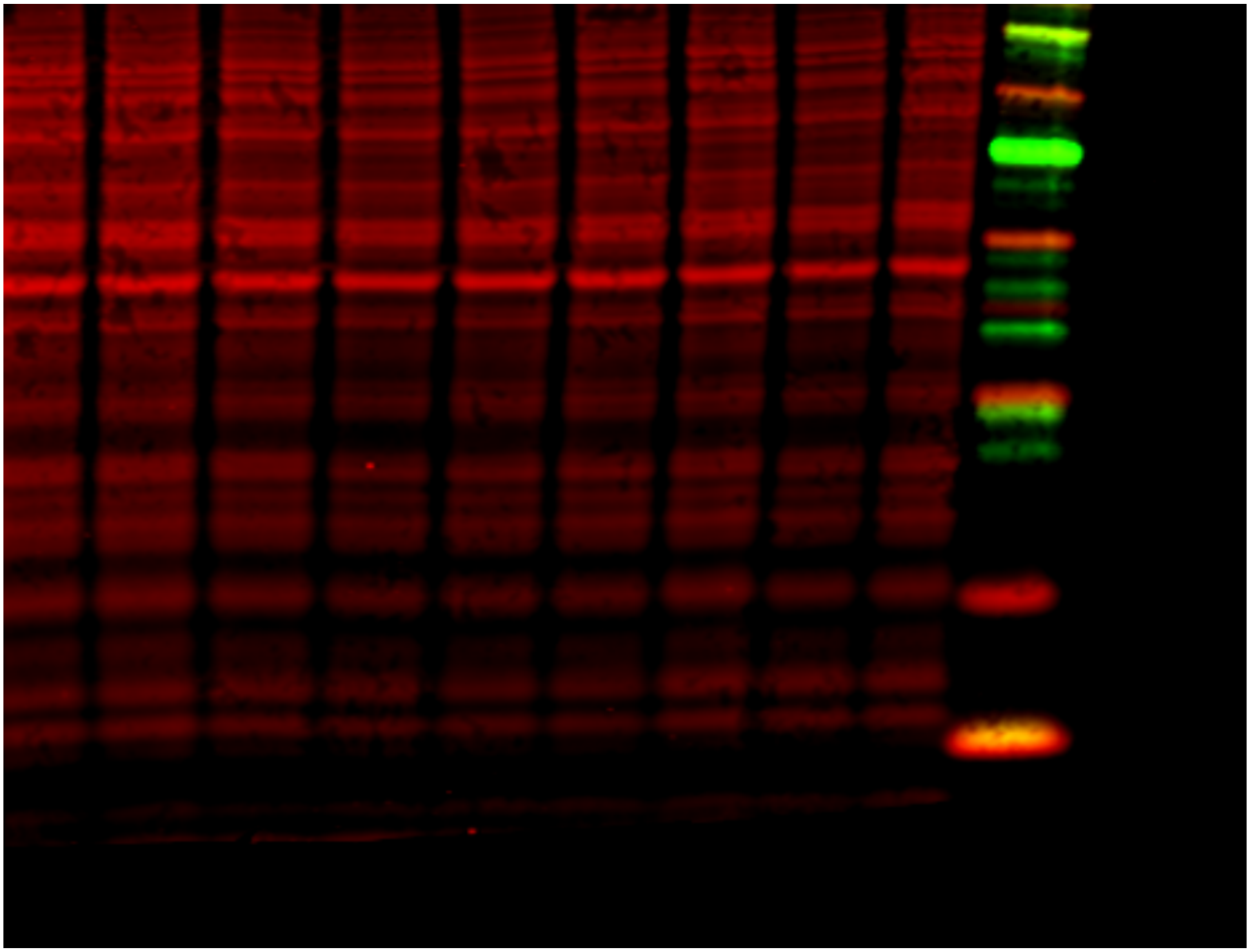

Fig S2C mitochondrial parkin

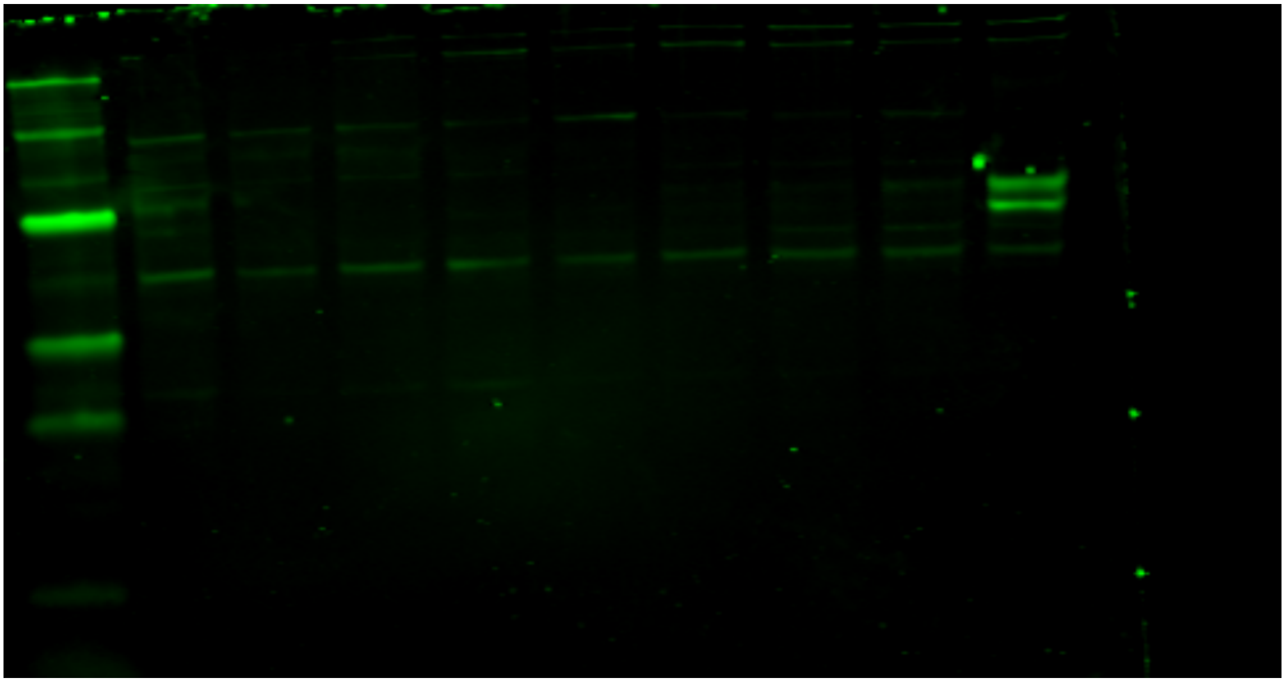

Fig S2C Mitochondrial total protein

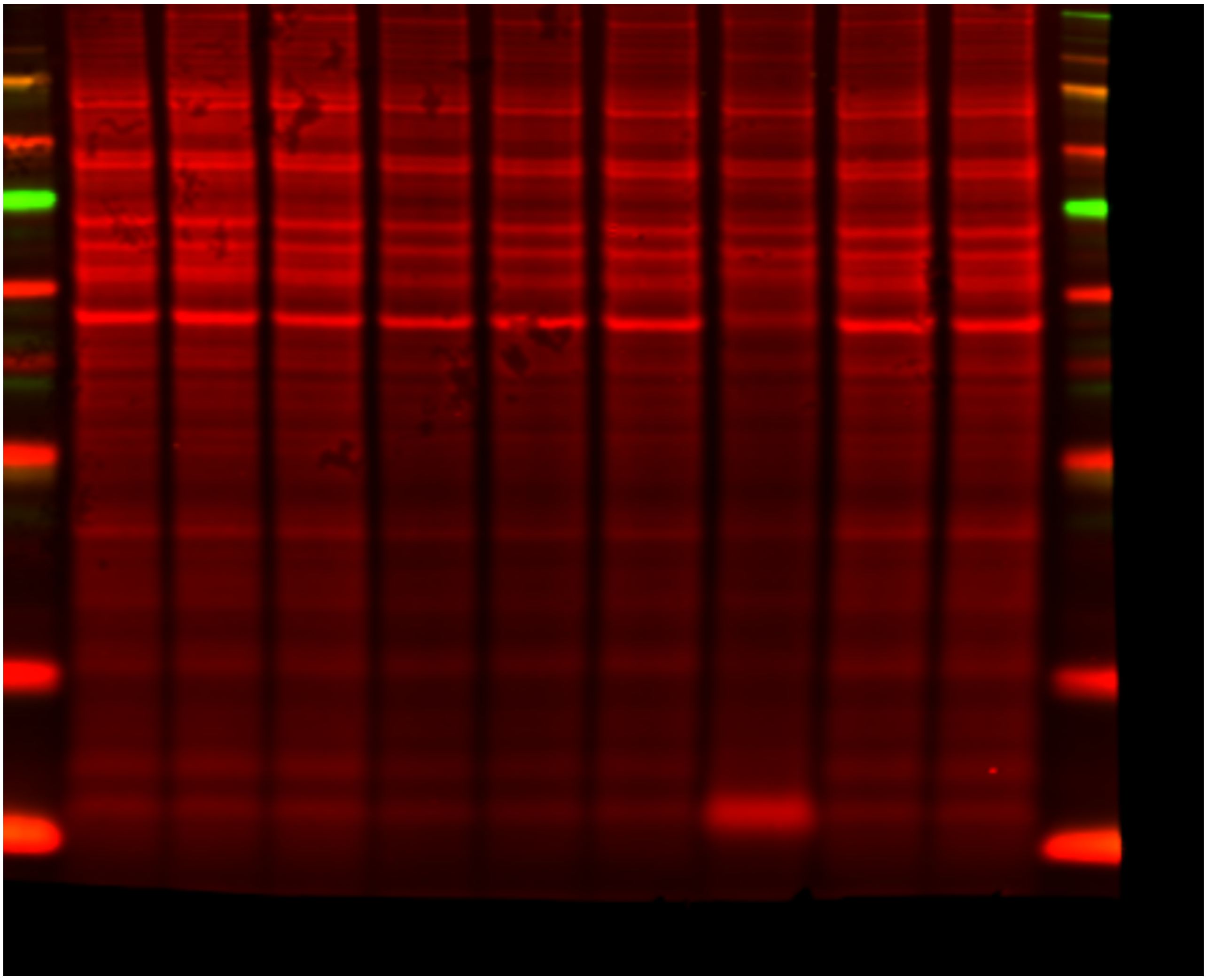

Fig S2C Whole Cell Parkin

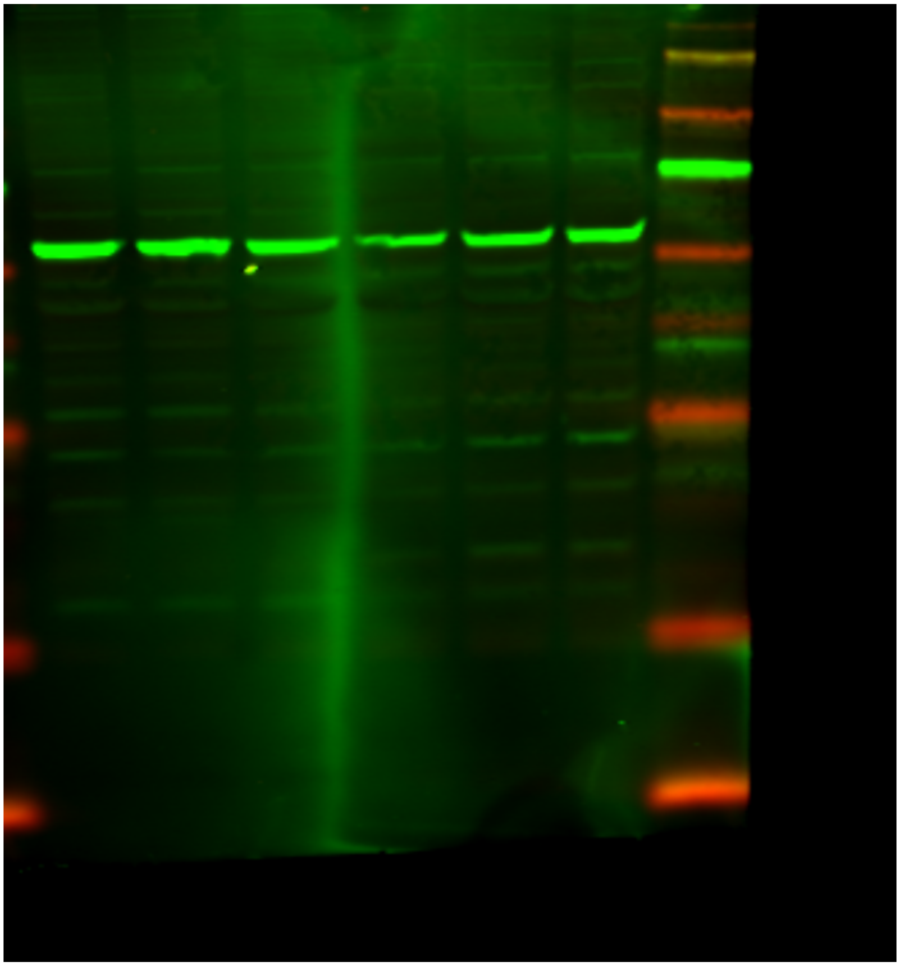

Fig S2C Whole Cell total protein

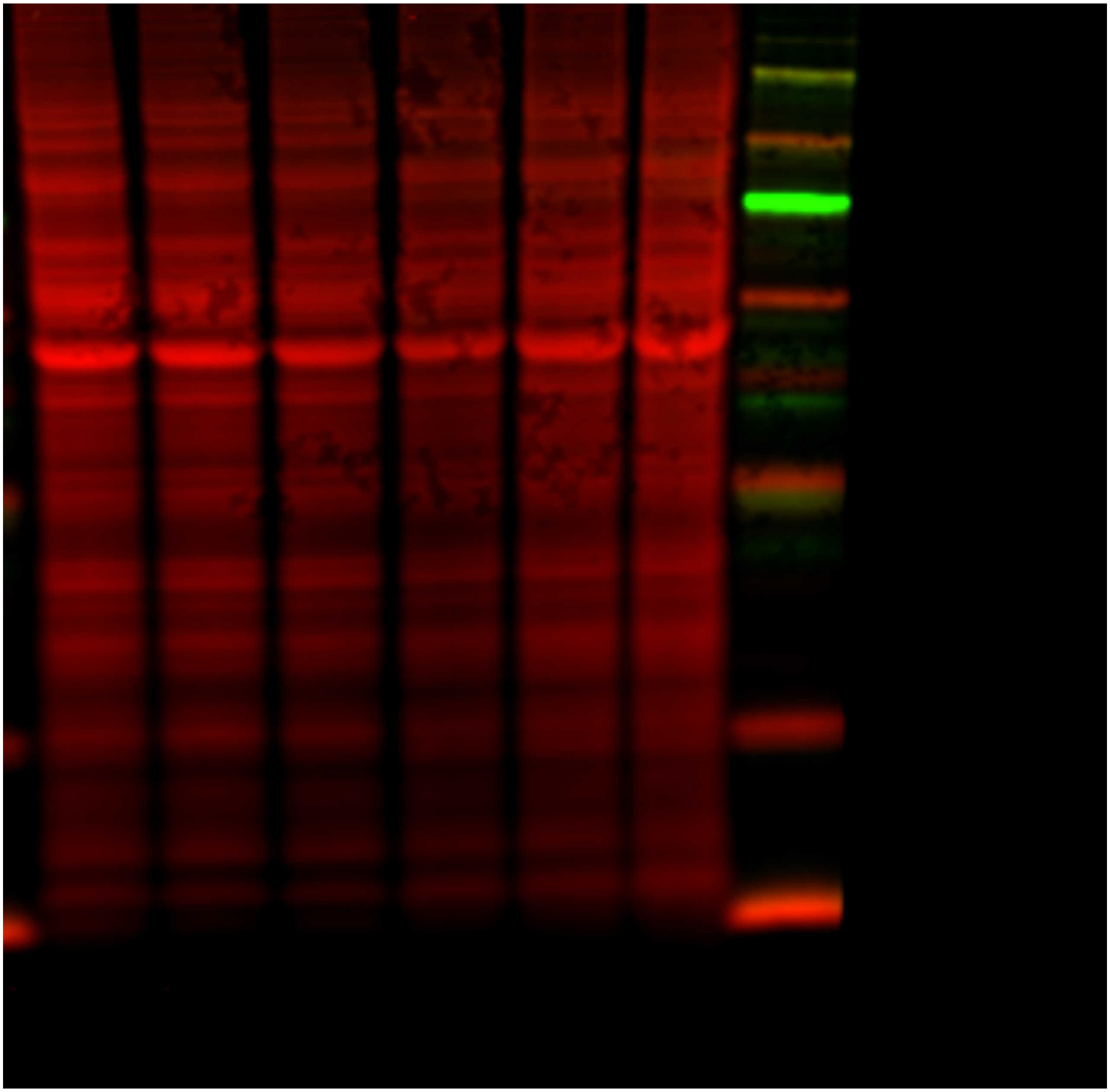

Fig S2D Cytoplasm Parkin

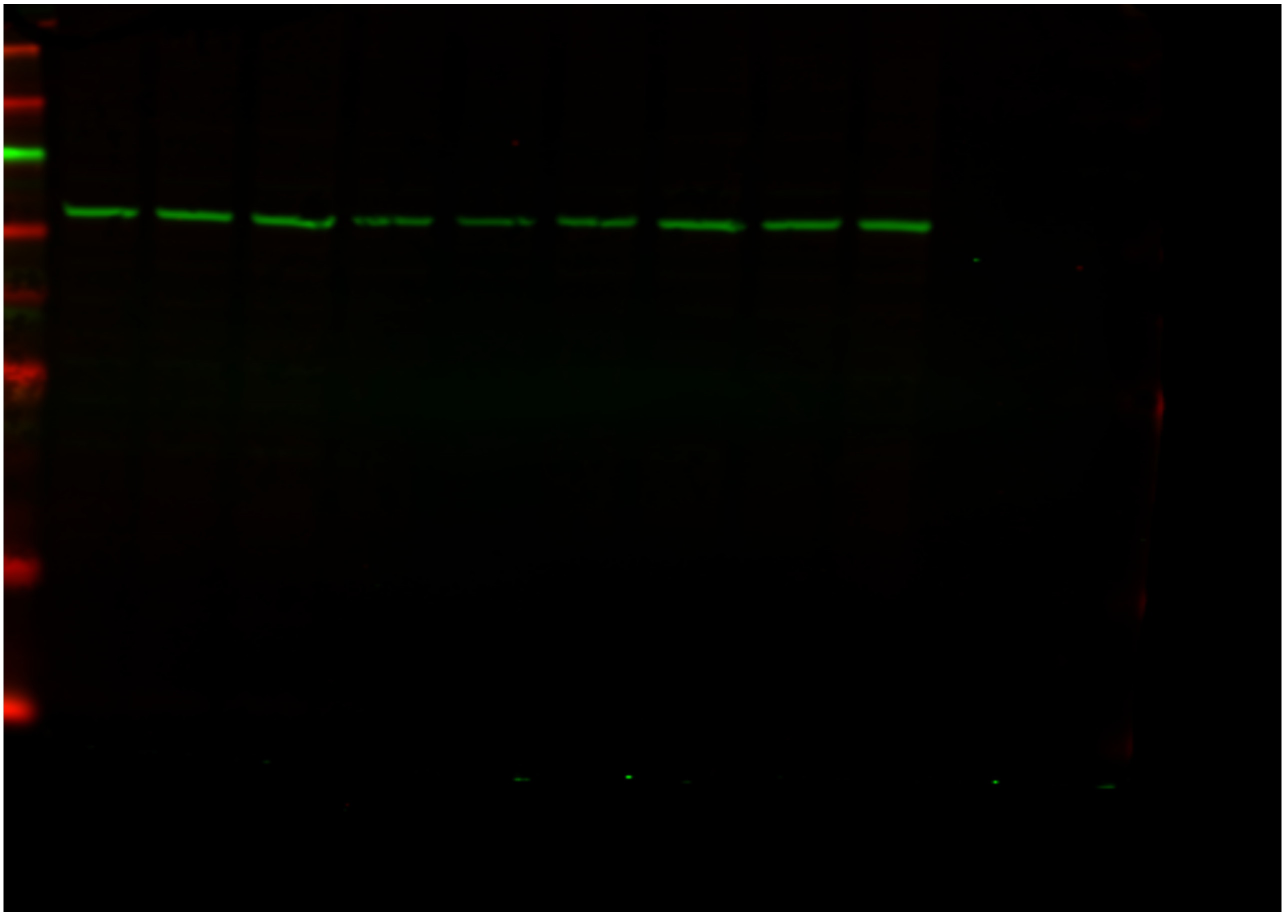

Fig S2D Cytoplasm total protein

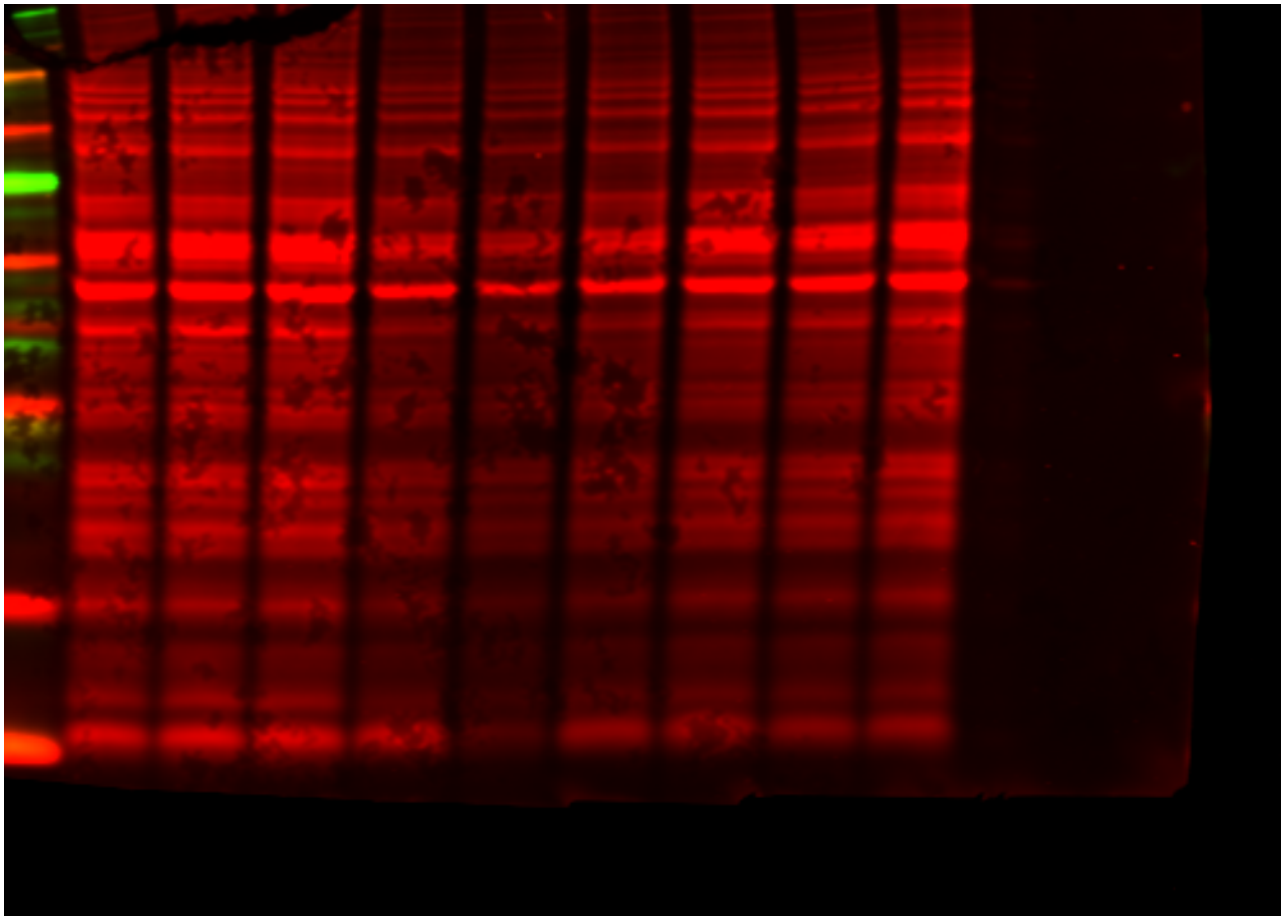

Fig S2D Mitochondrial Parkin

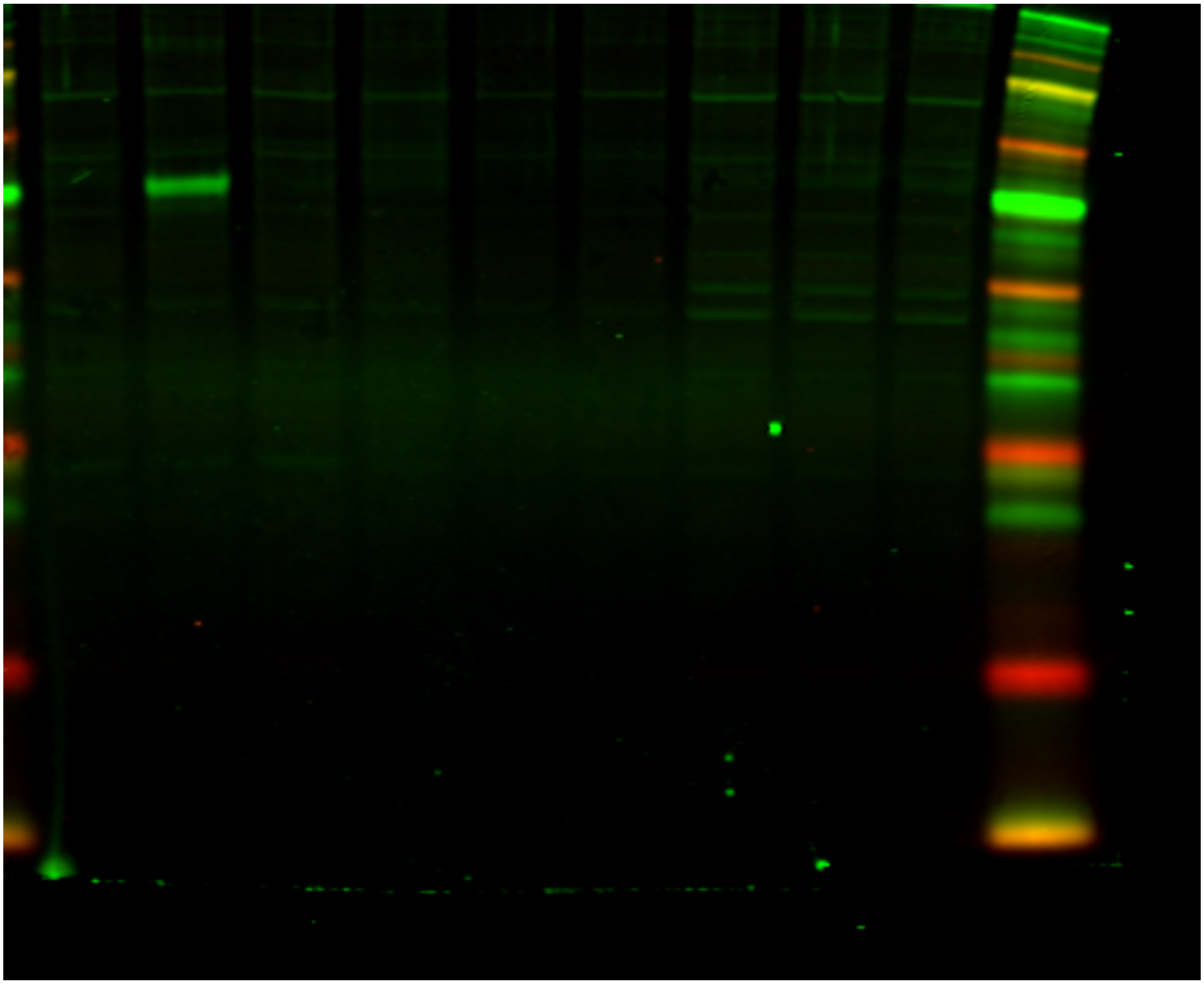

Fig S2D Mitochondrial total protein

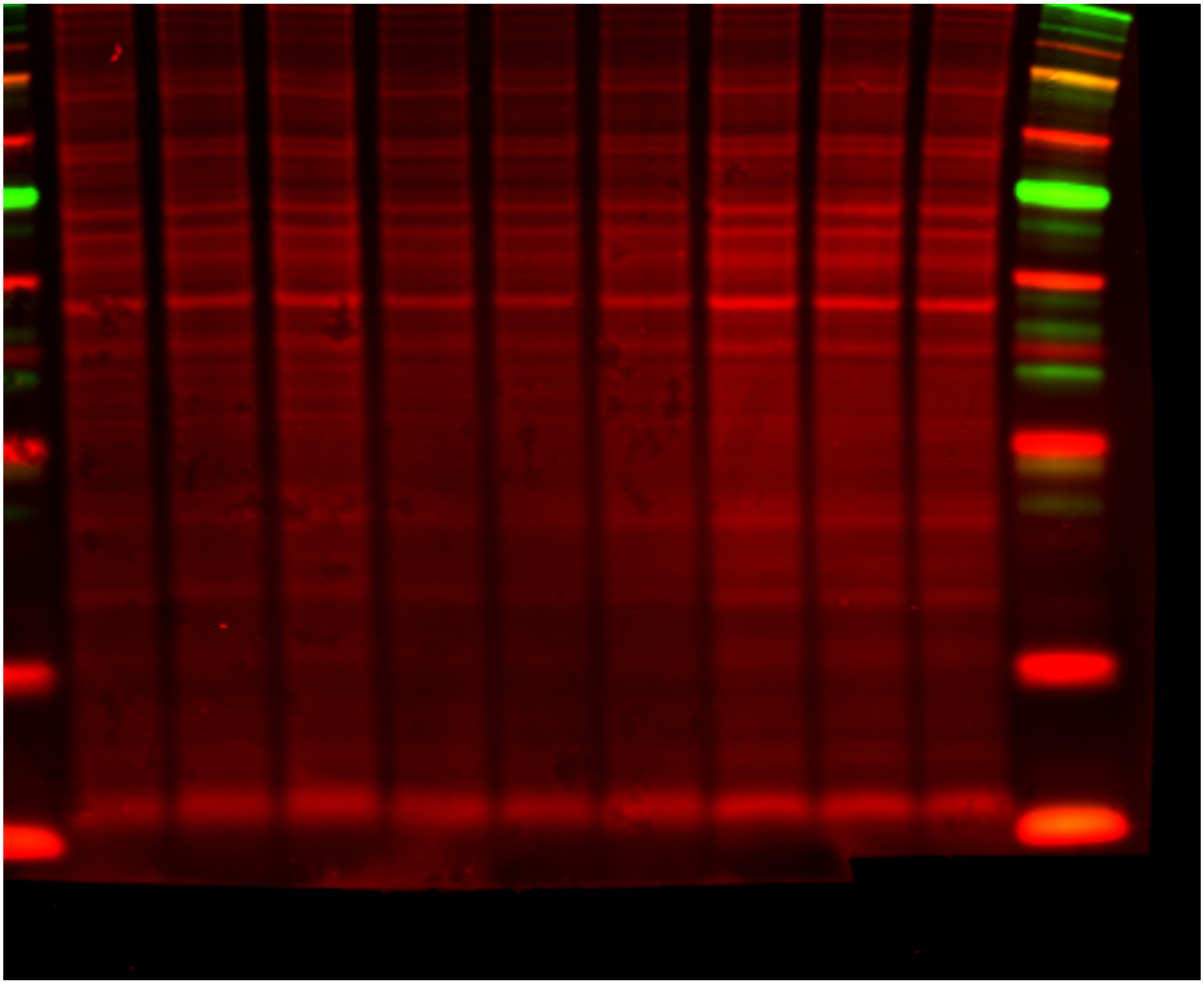

Fig S2D Whole Cell parkin

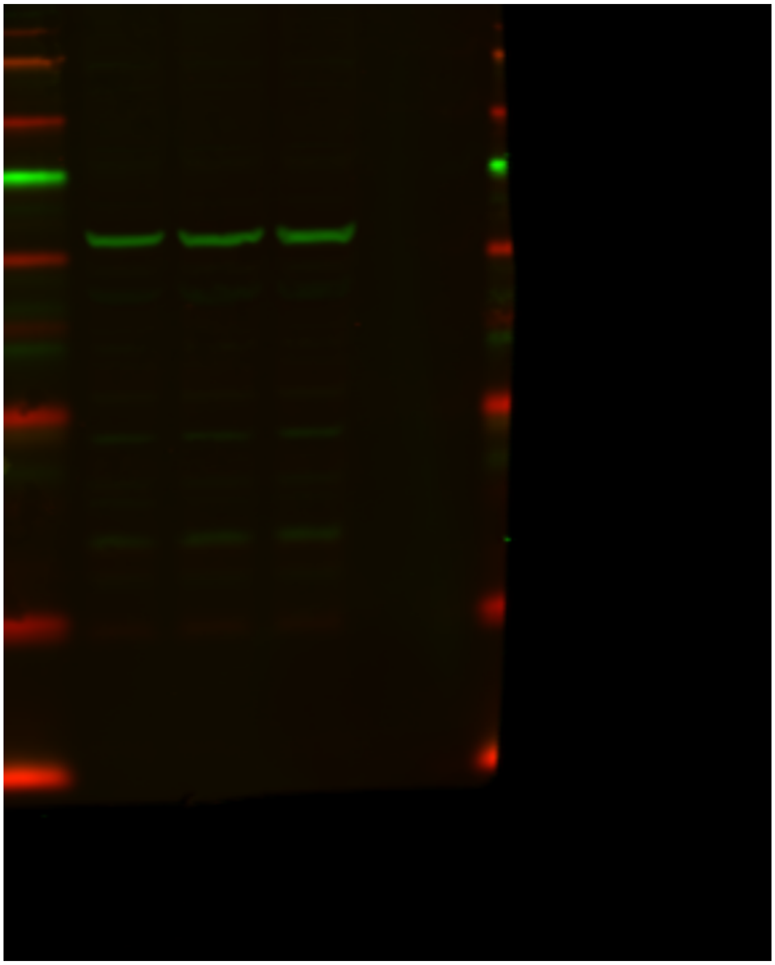

Fig S2D Whole Cell total protein

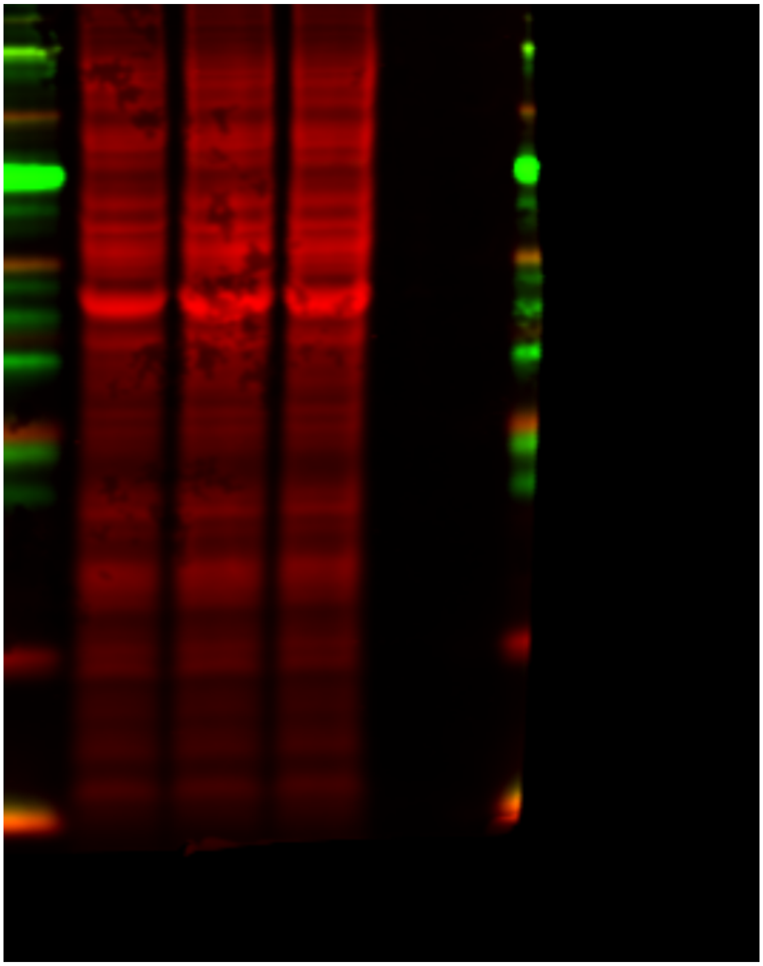

S1 Table. Inflammatory Cytokines and Receptors

Gene Symbol	Wild Type Fold Change	Wild Type p-Value	p53$^{-/-}$ Fold Change	p53$^{-/-}$ p-Value
Aimp1	5.78	**0.0170621**	10.63	**0.005045**
Bmp2	3.04	**0.0131283**	-1.34	**0.043676**
Ccl1	-1.84	**0.0027075**	1.35	**8.844E-05**
Ccl11	-1.35	0.0641538	1.14	0.0501585
Ccl12	22.59	**1.441E-05**	2.07	0.1263313
Ccl17	2.17	0.0512179	4.21	**0.0130904**
Ccl19	1.41	0.1143435	-1.77	**0.0006041**
Ccl2	42.47	**0.0013405**	-1.73	**0.0015438**
Ccl20	-1.95	**0.0001326**	1.33	**0.0063345**
Ccl22	1.67	**0.0108156**	-1.49	**0.007976**
Ccl24	8.39	**4.521E-05**	1.63	**0.0001133**
Ccl3	14.40	**3.421E-06**	3.01	**1.945E-05**
Ccl4	1.28	0.7207123	3.56	**0.0104619**
Ccl5	1.53	0.0515572	1.05	0.5984052
Ccl6	1.14	0.6335296	1.41	**0.000367**
Ccl7	-1.18	0.1571887	1.06	0.5833105
Ccl8	1.41	**0.0076416**	1.06	0.3590507
Ccl9	4.26	**2.997E-06**	1.43	**0.0004256**
Ccr1	1.50	0.0923223	1.55	0.2208175
Ccr10	12.06	**0.0038435**	3.71	**0.0128378**
Ccr2	-1.59	**0.0083674**	1.09	0.7939969
Ccr3	-1.18	0.4077851	1.68	**9.327E-05**
Ccr4	52.90	**1.548E-05**	3.11	**0.0105957**
Ccr5	3.71	**0.0357707**	3.53	**0.028034**
Ccr6	-1.64	**0.0076298**	-1.16	**0.0431875**
Ccr8	-2.09	**0.0009324**	1.49	0.0830078
Cd40lg	8.90	**8.041E-05**	1.18	0.3840235
Csf1	6.92	**0.0253222**	1.87	**0.0032036**
Csf2	1.66	0.3391881	2.55	0.0907316
Csf3	-1.38	**0.0004902**	-1.31	**1.523E-05**
Cx3cl1	1.52	0.5703898	1.39	0.5054915
Cxcl1	1.86	**0.0007185**	1.20	**0.0219124**
Cxcl10	26.36	**2.189E-06**	2.67	**1.84E-07**
Cxcl11	20.48	**2.944E-05**	-1.08	0.4227475
Cxcl12	1.96	0.2825518	1.41	0.7435229
Cxcl13	2.85	**0.0445671**	4.31	**0.0284849**
Cxcl15	-1.15	0.0965657	1.43	0.8075784

Cxcl5	2.10	**0.0266658**	2.87	**0.0004929**
Cxcl9	1.21	0.356626	-2.05	0.312987
Cxcr2	18.97	**0.0149768**	6.18	**0.0064784**
Cxcr3	-2.30	**0.0439571**	1.60	0.1332605
Cxcr5	1.19	0.6565017	1.09	0.5849043
Fasl	2.31	0.1505885	-3.97	**0.0152313**
Ifng	8.05	**0.0001493**	15.59	**0.0008562**
Il10ra	2.30	0.1012917	2.42	0.0944887
Il10rb	-1.09	0.3667979	1.38	**0.0055154**
Il11	-3.39	**4.056E-05**	1.03	0.9798409
Il13	-1.15	0.4518992	1.27	0.2398473
Il15	1.29	0.3039755	1.61	0.0136058
Il16	-1.29	0.3349479	1.05	0.7597522
Il17a	4.41	**0.0021836**	3.23	**0.0008986**
Il17b	3.60	**2.472E-05**	3.12	**0.002129**
Il17f	1.05	0.4576432	-1.76	**2.662E-06**
Il1a	1.13	0.6670768	1.66	0.2170615
Il1b	2.75	**0.0262681**	1.54	0.1637359
Il1r1	2.43	0.0961827	2.44	0.1061803
Il1rn	-1.32	0.7188125	**1.32**	0.4306608
Il21	4.10	**0.0128981**	1.94	**0.0488324**
Il27	-2.36	**0.0157364**	1.02	0.8695849
Il2rb	1.64	0.1660686	1.39	0.3696484
Il2rg	2.98	**0.0248864**	1.68	0.327176
Il3	8.03	**0.0006979**	7.64	**1.473E-05**
Il33	3.97	**0.0005581**	-1.54	**0.0028037**
Il4	2.92	**2.896E-07**	1.91	**1.453E-06**
Il5	1.56	0.0980721	1.04	0.6241751
Il5ra	1.88	**0.0268762**	1.22	**0.0373887**
Il6ra	2.02	0.2382262	2.25	0.1385329
Il6st	5.02	**0.0074599**	7.55	**0.0134946**
Il7	1.39	0.0912234	-1.15	0.2300767
Lta	1.64	0.2227227	3.35	0.116009
Ltb	2.91	0.0569834	1.66	0.1502654
Mif	-1.15	0.8223325	1.57	0.0729648
Nampt	3.31	**5.714E-08**	1.29	0.1354183
Osm	18.05	**2.946E-06**	2.36	**2.946E-06**
Pf4	-1.36	0.068677	1.13	0.096732
Spp1	-2.29	**1.206E-05**	-1.17	0.125186
Tnf	1.11	0.695727	1.51	0.0757056
Tnfrsf11b	-1.47	0.0701631	-1.15	0.2655547

Tnfsf10	-1.89	**0.0021014**	-1.17	0.406904
Tnfsf11	1.45	0.4056218	1.02	0.7812042
Tnfsf13	-1.13	0.6628014	-1.11	0.1773442
Tnfsf13b	-1.03	0.7753718	-1.08	0.2735128
Tnfsf4	-1.99	**3.759E-06**	-1.28	**0.0039736**
Vegfa	1.53	0.2772853	13.24	**0.0057169**

S1 Table. Inflammatory Cytokines and Receptors. This table contains the results for the full gene array. Red values in the "Fold-Change" column correspond to genes for each sample set that expression levels did not meet the limit of detection threshold. Bolded values in the "p-Value" column are statistically significant with a threshold set at $p \leq 0.01$

S2 Table. Extracellular Matrix and Adhesion Molecules.

Gene Symbol	Wild Type Fold Change	Wild Type p-Value	$p53^{-/-}$ Fold Change	$p53^{-/-}$ p-Value
Adamts1	-3.55	**6.686E-08**	-1.05	0.4038192
Adamts2	-1.22	0.1530748	-1.12	0.2546388
Adamts5	-4.36	**8.055E-07**	-1.34	0.1095027
Adamts8	3.34	**0.0003422**	2.91	**0.0368682**
Cd44	1.84	**0.0010165**	1.31	0.1082477
Cdh1	2.64	0.9559081	2.12	0.1523006
Cdh2	12.02	0.2134534	-1.67	**0.005067**
Cdh3	-3.92	**0.0003848**	-2.80	**0.0023096**
Cdh4	5.34	**0.0051882**	3.37	0.1091118
Cntn1	2.43	0.5095791	1.31	0.0500058
Col1a1	-2.60	**5.299E-05**	-1.26	0.338308
Col2a1	-1.02	0.7415428	-1.64	**0.0330664**
Col3a1	-1.88	**0.0001075**	-1.57	**0.0260698**
Col4a1	-1.99	**4.153E-06**	-1.28	0.0800652
Col4a2	-3.05	**2.053E-06**	-1.25	0.3204889
Col4a3	-4.45	**0.0001849**	-1.15	0.9443072
Col5a1	-3.45	**1.06E-09**	-1.57	**0.0406681**
Col6a1	-2.47	**1.57E-06**	-1.30	**0.0446823**
Ctgf	1.13	0.1143722	-1.16	0.2804366
Ctnna1	1.07	0.2121615	1.10	0.1014728
Ctnna2	-2.98	**2.486E-05**	-1.39	0.6589596
Ctnnb1	-1.17	0.1436711	-1.18	0.2392513
Ecm1	2.25	**0.0004105**	1.25	0.1529486
Emilin1	-3.04	**5.64E-08**	-1.58	**0.0206557**
Entpd1	1.13	0.3544631	1.45	**0.0391311**
Fbln1	-1.09	0.2566525	-1.43	0.1097958
Fn1	-2.29	**8.632E-07**	-1.38	**0.0252431**
Hapln1	-3.67	**0.0267599**	-2.42	0.0703831
Hc	1.86	**0.0057445**	4.08	**0.0234426**
Icam1	2.87	**2.837E-05**	1.13	0.4380066
Itga2	1.55	**0.0016469**	1.06	0.5586151
Itga3	-1.09	0.3323105	-1.01	0.9561112
Itga4	-2.33	**2.371E-06**	-1.08	0.4640974
Itga5	-1.26	**0.0137164**	1.04	0.5479389
Itgae	-1.04	0.6009413	1.29	0.9885051
Itgal	-3.73	**9.66E-07**	-1.14	0.2312796
Itgam	-2.13	**0.0003203**	-1.30	0.1565445

Itgav	-1.47	0.000349	-1.07	0.5060534
Itgax	-2.62	**7.668E-06**	1.15	0.3015896
Itgb1	-1.26	**0.0003651**	-1.01	0.9121545
Itgb2	-1.85	**0.0007792**	-1.13	0.4160693
Itgb3	1.01	0.8331778	-1.17	0.252719
Itgb4	1.21	0.2141892	1.02	0.8883886
Lama1	-7.72	**5.152E-06**	-1.75	0.1372408
Lama2	-2.81	**5.068E-06**	-1.15	0.1541637
Lama3	5.67	**0.0001698**	8.30	**0.0100523**
Lamb2	-1.16	0.177013	1.04	0.555006
Lamb3	5.38	**0.0013964**	1.44	0.1527717
Lamc1	-3.13	**2.626E-06**	-1.56	**0.0165854**
Mmp10	12.44	**2.847E-07**	2.45	0.2229929
Mmp11	-1.29	**0.0018195**	-1.05	0.640219
Mmp12	3.38	**0.0007145**	1.76	0.1488446
Mmp13	4.55	**8.948E-07**	2.87	0.0928837
Mmp14	-1.68	**0.007121**	-1.01	0.9127375
Mmp15	24.41	**7.796E-05**	1.63	0.3369645
Mmp1a	1.12	0.6316478	1.02	0.9476694
Mmp2	-1.27	**0.0233539**	**1.04**	0.3857575
Mmp3	2.97	0.242328	-1.06	0.7272636
Mmp7	1.52	0.1679582	1.18	0.8315208
Mmp8	7.53	**4.347E-07**	1.23	0.3783723
Mmp9	-1.04	0.9698434	1.18	0.7253632
Ncam1	-2.74	**1.26E-05**	-1.55	0.1314947
Ncam2	7.23	**3.863E-05**	1.74	0.3753636
Pecam1	-2.93	**5.258E-08**	-1.39	0.166696
Postn	-1.35	**0.0007211**	-1.35	**0.0119482**
Sele	1.94	**0.0032174**	1.35	0.1240251
Sell	2.65	0.0512159	1.66	0.3959163
Selp	1.07	0.246202	-1.37	0.0566941
Sgce	1.95	**0.0405941**	1.01	0.7665848
Sparc	1.16	0.152223	-1.31	**0.0188153**
Spock1	8.09	**0.0015725**	2.77	0.0824143
Spp1	-1.22	0.2727248	-1.01	0.8774079
Syt1	5.33	**0.0235928**	6.00	0.1530125
Tgfbi	-1.02	0.7726811	-1.11	0.3677866
Thbs1	-1.17	0.103955	-1.12	0.0775988
Thbs2	-1.60	**7.774E-05**	-1.38	0.1696684
Thbs3	1.33	**0.0215896**	-1.01	0.7457941
Timp1	1.57	0.064872	1.14	0.1411936

Timp2	1.11	0.3381153	1.21	0.0579834
Timp3	-2.18	**0.0003166**	-1.24	**0.0401915**
Tnc	-1.38	**0.0370964**	-1.31	0.098737
Vcam1	2.46	**1.997E-06**	1.26	0.0930905
Vcan	-1.08	0.3837069	-1.53	**0.0164413**
Vtn	6.67	**0.0009301**	4.19	0.0947995

S2 Table. Extracellular Matrix and Adhesion Molecules. These tables contain the results for the 86 genes on each array. Red values in the "Fold-Change" column correspond to genes for each sample set that expression levels did not meet the limit of detection threshold. Bolded values in the "p-Value" column are statistically significant with a threshold set at $p \leq 0.01$
