# Supplementary material for: Doxorubicin-induced p53 interferes with mitophagy in cardiac fibroblasts
Source: PLoS One. 2020 Sep 22;15(9):e0238856. doi: 10.1371/journal.pone.0238856 (PMC7508395; doi:10.1371/journal.pone.0238856)
Supplement: Attachment — Submitted filename: Response to Reviewers.pdf [file pone.0238856.s004.pdf]

Dear Reviewers:

Thank you for the thoughtful and clear critiques of our manuscript. Below we have responded to each of the points raised. For clarity, each review point is included in black text and our response in blue text.

Reviewer #1:

The current study "Doxorubicin-induced p53 Interferes with Mitophagy in Cardiac Fibroblasts" discussed the effect of p53 deficiency on Doxorubicin induced mitochondrial change. Here are some major concerns:

1. The authors need to better arrange the data presentation and result description. Many closely related figures should be combined together. Conclusions in each part should be clearly given. Also, the authors should be more careful about the figure number and panel consistency. Figures have been combined to reduce the overall number of figures and careful attention was paid to the panel labeling in the construction of the figures. Conclusion statements were added to the results sections. Through this process we have taken the 13 figures in the original submission and condensed them to 7 primary figures with two supplementary figures.

2. The current study points out that DOX compromises mitochondria in the wild type cells but not the p53 deficient cells. More than half of the data are the consequences of this phenotype, including ROS, metabolism change and mitophagy increase in the wild type cells. Have the authors researched into the reason p53 deficient cells are resistant to the DOX mediated mitochondria damage? While there are most likely multiple mechanisms related to the $p53^{-/-}$ attenuation of DOX toxicity in CFs, we explored a specific mechanism wherein p53 upregulation from DNA damage and cellular stress interferes with physiological and pathological mitochondrial quality control. Mitochondrial quality control includes the process of Parkin-dependent mitophagy. We found that p53 sequesters Parkin in the CFs preventing the clearance of dysfunctional mitochondria through the mitophagy process. In the absence of this sequestration by p53, Parkin is free to mediate removal of dysfunctional mitochondria and results in the phenotype noted in p53-deficient cells.

3. The authors found that DOX treated p53 deficient cells have similar proliferation phase pattern compared to the non-treated mice, which is an interesting phenotype compared to WT cells. However, DOX really inhibits the cell proliferation in both cells. Have the authors discussed the mechanism? The possible mechanism is only briefly discussed in this manuscript as it does not appear to be related to p53 cell cycle regulation in the $p53^{-/-}$ cells. For this manuscript we wanted to focus more on the changes in mitophagy.

4. Western blot in the same assay with all samples is needed to confirm the upregulation claim of Parkin in p53 deficient cells. This is now included in supplemental figure S2B

5. “Difficult to assess” and “not three repeats” should not be used to cover the unshown or unexplained data This data was moved to the supplemental section and that language removed.

6. If Parkin is not upregulated in the WT cell mitochondria in DOX treatment, how do the authors explain the upregulated mitophagy? Mitophagy is not upregulated in the WT cells after exposure to DOX. The level of mitophagy is similar to that of the WT control cells.

7. In figure 12, the interaction of Parkin and p53 is mostly in the nucleus. How is it related to

mitophagy? Also, FCCP treatment should be put in Figure 12 too. Both p53 and Parkin are present in the cytoplasm but can cross the nuclear membrane to act as transcription factors. Additionally, under cell stress, mitochondria often take on a perinuclear localization pattern. The p53/Parkin interaction may be taking place inside the nucleus or just outside the nuclear membrane. Importantly, our data shows that this interaction does occur and is increased after DOX exposure. We did not include FCCP in Figure 12 from the original manuscript because our experiments indicated that it does not robustly induce p53 expression.

8. Judging from the current data, p53 deficiency protects the CF cells from the very beginning of DOX treatment. The authors need to make the conclusions more carefully. We took this comment into account and throughout our revisions made a point to draw our conclusions based upon the data we present and to not go beyond these included experiments.

Reviewer #2

1. The authors used isolated fibroblasts from WT and P53 deficient mouse for in vitro experiments. It should be better to check the Dox-induced cardiac toxicity and involvement of p53 pathway in vivo. If the in-vivo datas are included with evaluation of DNA damage, mitochondrial dysfunction, cell cycle arrest, cell death on cardiac cells after Dox IP injection on WT and P53 KO mice, that will strengthen the authors' ideas. p53 KO mice cannot live beyond 3 months without developing tumors, due to full body knockout of this critical tumor suppressor gene. We isolated cardiac fibroblasts before exposing them to DOX to study the role of p53 solely in CFs. We chose to perform these initial studies *in vitro* to test our hypothesis. Our lab is currently developing mice that have an inducible KO of p53 that will only be present in cardiac fibroblasts. Using these mice important questions such as the point raised here can be addressed. However, for this manuscript we felt these experiments were beyond the scope of our initial efforts to establish DOX-induced mitochondrial damage in cardiac fibroblasts as a damage mechanism with the potential to cause cardiac pathology.

2. The authors described the results obtained in figure legends. In figure legend is only for demonstrating the methodology how to conduct experiment. Please re-phase figure legends. Figure legends were modified to only reflect methods.

3. Table1 and 2, it will be better to re-organize these tables to hitmap. And try to merge Table 1 and Table 2 for easy understanding and after merging re-organize merged information to hitmap. Attempts were made to combine and reconfigure the data into a heat map that was intuitive to read. However, we were not satisfied that the end products were useful. Since many of the genes that were differentially regulated differed between the WT and KO mice, we did not combine the tables in their current formats. In our opinion, we believe that presenting this data in concise table format is superior because we can present the exact fold change in a way that is easy to view quickly.

4. In Figure2B & C, it is recommended to show actual FACS plot data (including the percentage of G1,S and G2) for better understanding at a glance. This figure was kept "as is" because we prefer to show the average and SEM for the biological replicates. This is not an uncommon way to show this data as this type of depiction is frequently found in the scientific literature.

5. There is too much information in Materials & Methods. It will be better to make compact. Revisions for clarity and brevity were made where possible. A major contributor to the overall section length is the large number of different techniques used in the work we present.

6. There are some minor errors/mistakes in manuscript and figures. Please re-check them very carefully and thoroughly. Some identified was shown below.

- In Figure 1, panel C and D are missing. Figure 1 has been revised to include more panels. Panel labeling is now correct.

- In Figure3, put “WT” on the top of Panel A,C,E column and put “P53-/-” on the top of Panel B,D,F column. Suggestion taken and changes made.

- In Figure4C, the cell number between groups is too different. Show the representative images which have similar cell number. The difference in the number of cells is due to the different cellular growth rates of the different strains. We felt it was more important to keep the seeding density, growth time, and magnification unchanged between the samples.

- In Figure5A and B, Put “mitotracker” and “JC-1” on the top of panel A and B for better understanding. Suggestion taken and changes made.

- In Figure5C, put the full name of MTG to mitotracker green for easy understanding. Suggestion taken and changes made.

- In Figure6 and manuscript, the authors used terms “baseline” and “stressed”. It might be better to change “con” and “Dox”. Baseline and stressed have different meanings than control and DOX. In this experiment, cells were exposed to DOX (or not) and during assessment mitochondrial stressors were added. Baseline and stressed refer to before and after the addition of mitochondrial stressors.

- In Figure9B and C, it will be better to move to supporting data. Suggestion taken and this figure is now supplemental Figure S1.

- In Figure 10A, it will be better to move to supporting data. Suggestion taken and the figure is now supplemental Figure S2.

- In Figure 10C, graph (mitochondrial) is missing. This data is now included supplemental data due to the fact that the results were observed and not quantified.

- In Figure11, indications for A and B are missing. Panels are now labeled.

- Page 9, paraformaldehyde, put the % of paraformaldehyde (might be 4%). The % formaldehyde was added to the methods section.

- Page11, what is the pore size of Boyden chamber? The pore size was added to the methods sections.

- Page 15 line11, what is the dose of DOX? All figure legends were reviewed to ensure dosing information is included.
