# Supplementary material for: Doxorubicin-induced p53 interferes with mitophagy in cardiac fibroblasts
Source: PLoS One. 2020 Sep 22;15(9):e0238856. doi: 10.1371/journal.pone.0238856 (PMC7508395; doi:10.1371/journal.pone.0238856)
Supplement: Attachment — Submitted filename: Response to Reviewers.pdf [file pone.0238856.s005.pdf]

Dear Reviewers:

Thank you for the thoughtful and clear critiques of our resubmitted manuscript. Below we have responded to each of the points raised. For clarity, each review point is included in black text and our response in blue text.

1. The authors made the main conclusion that p53 hindering the Parkin localization to mitochondria is the culprit of insufficient mitophagy and mitochondrial dysfunction. However, there is no direct evidence to the mitophagy of p53 deficient cells because result of Figure 4E failed. The authors should perform the same experiment in Figure S1 in p53 deficient cells and compare with the WT cells.
   To provide further clarification we would respectfully point out that the results did not fail. Gating for positive/negative lysosomal cells and positive/negative mitophagy cells was done under the supervision of the UTHSCSA Flow Cytometry Core using WT samples (control, DOX, FCCP) without dyes and with each dye separately. The intensity of each dye was maximally increased. The increased intensity in the mitophagy dye alone demonstrates the increase in the process. It is the acidification/intensification of the mitophagy dye that demonstrates the fusion of the phagosome with the lysosome. Due to the excess amount of Parkin at baseline, the majority of the $p53^{-/-}$ cells in each sample group were undergoing maximal mitophagy. Overall, this demonstrates that in the absence of p53, these cells are able to undergo mitophagy. The mitophagy assay was repeated in the $p53^{-/-}$ cells and visualized with fluorescent microscopy. In our revision, this experimental data was added as panel B in Fig S1. The middle panel of Fig S1B is an example of the mitophagy dye in the $p53^{-/-}$ cells. Compared to the control cells of the WT cells, a significant increase in the amount and intensity of the red fluorescence can be seen in every treatment group of the $p53^{-/-}$ cells. The fluorescence of the green lysosome dye is also increased in each treatment group.

2. According to Figure 6 and Figure S2, Parkin binds to p53 so that it cannot localize to the mitochondria and induce mitophagy after DOX treatment. Why would the staining of Figure 6 show a granule pattern? Are they on mitochondria?
   Figure 6B and 6C are not indicative of a granular staining pattern per se, but rather the appearance related to the methodology used to visualize the interaction. The fluorescence arises from the proximity of complimentary DNA probes that are ligated together and then amplified with fluorescent oligonucleotides. There are multiple examples from the literature that demonstrate the fluorescent pattern with the proximity ligation assay. However, considering the proximity that transcription factors like p53 and Parkin have to the nucleus it would not be surprising that the interactions of the two proteins would be near the nucleus and/or mitochondria. Moreover, Parkin has the additional function of regulating mitophagy via its translocation between the mitochondria and cell nucleus. Both the results and discussion have been revised to further clarify these considerations.

   Support from the literature (references added to manuscript)
   - Alves de Costa: https://www.ncbi.nlm.nih.gov/pmc/articles/PMC6341214/
   - Al-Mehdi: https://www.ncbi.nlm.nih.gov/pmc/articles/PMC3565837/
   - Park: https://www.ncbi.nlm.nih.gov/pmc/articles/PMC125431/

3. In figure 1ABC the WT and p53 deficient cells both showed suppressed growth treated with DOX. However, in figure 1GJ, the cell cycle pattern is much better for p53 deficient cells. The authors need to provide some explanation why the p53 deficient cells do not show much better growth than WT cells under DOX treatment. The decreased proliferation seen in the $p53^{-/-}$ is likely due to a combination of things. DOX alters gene expression. One study demonstrated repression of pathways normally activated in the G1 phase of the cell cycle. These pathways are integral to the "doubling" of cell contents necessary for proliferation. Inhibiting mitophagy can often lead to apoptosis, likely via cell stress pathways including p53-mediated cell cycle arrest. However, inducing mitophagy has been shown to decrease cell proliferation simply due to the high energy demand of mitophagy and mitochondrial biogenesis.

   Support from the literature (references added to manuscript)
   - Nikerel: https://www.ncbi.nlm.nih.gov/pmc/articles/PMC6135803/
   - Boyle: https://www.jbc.org/content/293/38/14891.short
   - Pang: https://www.ncbi.nlm.nih.gov/pmc/articles/PMC3674280/
   - Nanasi: https://journals.plos.org/plosone/article?id=10.1371/journal.pone.0231223
   - Kang: https://www.tandfonline.com/doi/full/10.1080/21691401.2019.1593854
   - Wei: https://link.springer.com/article/10.1007/s12192-018-0937-7
   - Niu: https://www.sciencedirect.com/science/article/pii/S1382668916301442

4. In figure 2A, the p53 deficient cells have similar pattern of ROS production. p53 deficient cells has higher ROS than WT cells. The following data all address the better mitochondrial function in p53 cells. The authors need to discuss the possible source of ROS and the reason p53 deficient cells do not get more damage.
   Figure 2A addresses ROS production in the entire cell, not just at the mitochondria. Figure 2B shows that there is a greater variability in the production of ROS associated with the mitochondria in p53 null cells, whereas mitochondrial ROS is more uniformly increased in WT cells. The variability of mitochondrial ROS production of p53 null cells could reflect the process of mitophagy, as dysfunctional ROS-producing mitochondria are degraded. Additionally, both groups are relative to their control baseline. Since it appears that mitophagy is increased at baseline in the p53 null cells, the baseline mitochondrial ROS production could be decreased as mitochondria are more efficiently cleared. DOX has multiple mechanisms of producing ROS in cells. Some of these are mediated via mitochondria and mitochondrial dysfunction. However, DOX can create free radicals by interacting with $Fe^{2+/3+}$ in the cell. The cellular increase of ROS seen in WT and p53 null cells is a combination of DOX/Fe and mitochondrial production. The scope of our paper is limited to the ROS produced by mitochondria and the resulting mitochondrial dysfunction. Other organelles of the cell are also damaged by the increase in cellular ROS and we did not study the effect of p53 on these other organelles. It is likely that there are many significant changes in the p53 null cells that are beneficial to the other organelles.

Support from the literature (references added to manuscript)

- DOX ROS production: https://clincancerres.aacrjournals.org/content/20/18/4737 ; https://www.sciencedirect.com/science/article/pii/S0167488916300118
- Nuclear damage: https://www.pnas.org/content/117/26/15182
- Golgi damage: https://www.ncbi.nlm.nih.gov/pmc/articles/PMC3374628/
- ER damage: https://pubmed.ncbi.nlm.nih.gov/31580970/ ; https://pubmed.ncbi.nlm.nih.gov/26838784/
- Membrane damage: https://pubmed.ncbi.nlm.nih.gov/29019935/ ; https://journals.plos.org/plosone/article?id=10.1371/journal.pone.0026441

5. If the bizarre result of Figure 4E is because of their difficulty to collect reliable data from p53 deficient cells, please delete the result. It is hard to believe every single mitochondria is going through mitophagy in p53 deficient cells in any condition. Also, Parkin abundancy is not sufficient to explain the authors' difficulty getting the data. Parkin is not directly related to the method in this experiment. The authors need to thoroughly discuss why they cannot get the exact percentage of mitochondria going through mitophagy in p53 deficient cells.
   The discrepancy comes from measuring mitophagy in cells and not individual mitochondria. WT samples were used to define the population of Mito+, Lyso+, and "double positive" cells based on the amount of fluorescence. 98-100% of the cells from the p53 null samples showed max fluorescence with the lysosome dye and the mitophagy dye. Additional comments on this population have been added to results section.

6. p53 deficiency was introduced by deleting exons 2-6, which includes the translation starting site, not the promoter. The authors need to correct the description of this gene modulation.
   Corrected in the manuscript

7. In Figure S2D, mitochondrial Parkin western blot should be statistically analyzed. It is also hard to see a molecular weight shift up. Also, if FCCP exerts its action without the involvement of p53, why would we not see a similar band of Parkin in the p53 deficient mitochondria in the FCCP group?
   This figure was moved to the supplemental section at the suggestion of the reviewers, as opposed to removing it from the paper completely. The MW shift up can be seen in S2C where the lighter Parkin bands can be seen in each treatment group in the mitochondrial fraction. It is a small MW shift, indicative of an addition of only 5-10 kDa. There should be a Parkin band in FCCP-treated sample in the mitochondrial fraction of the p53 null cells. This band was seen in other blots, but the band was not seen in the DOX-treated cells. We believe this is due to the instability of the modified protein. Samples in which the band was previously seen would be rerun on the same blot for quantification and imaging, but not appear this time.

8. The authors need to describe Figure 5B in the results part. Why is p53 found in these cells? What is the pattern of Parkin staining?

The diffuse, faint staining seen in figure 5B is not specific to p53. The puncta of the fluorescence does not have the same intensity as that seen in the WT examples. This diffuse patterning also appears to “shade” in the shape of the cell. This background fluorescence is also slightly visible in the WT cells. The contrast of the images were adjusted together so that the p53 staining could be seen when merged with the Parkin images. This adjustment in contrast enabled in the visualization of the background fluorescence. Figure 5B is addressed more specifically in the text now.
